# Whole-genome CpG-resolution DNA Methylation Profiling of HNSCC Reveals Distinct Mechanisms of Carcinogenesis for Fine-scale HPV+ Cancer Subtypes

**DOI:** 10.1158/2767-9764.CRC-23-0009

**Published:** 2023-08-30

**Authors:** Tingting Qin, Shiting Li, Leanne E. Henry, Elysia Chou, Raymond G. Cavalcante, Bailey F. Garb, Nisha J. D'Silva, Laura S. Rozek, Maureen A. Sartor

**Affiliations:** 1 Department of Computational Medicine and Bioinformatics, University of Michigan Medical School, Ann Arbor, Michigan.; 2 Department of Periodontics and Oral Medicine, School of Dentistry, University of Michigan, Ann Arbor, Michigan.; 3 Department of Pathology, University of Michigan Medical School, Ann Arbor, Michigan.; 4 Rogel Cancer Center, University of Michigan, Ann Arbor, Michigan.; 5 Department of Environmental Health Sciences, School of Public Health, University of Michigan, Ann Arbor, Michigan.; 6 Department of Biostatistics, School of Public Health, University of Michigan, Ann Arbor, Michigan.

## Abstract

DNA methylation is a vital early step in carcinogenesis. Most findings of aberrant DNA methylation in head and neck squamous cell carcinomas (HNSCC) are array based with limited coverage and resolution, and mainly explored by human papillomavirus (HPV) status, ignoring the high heterogeneity of this disease. In this study, we performed whole-genome bisulfite sequencing on a well-studied HNSCC cohort (*n* = 36) and investigated the methylation changes between fine-scaled HNSCC subtypes in relation to genomic instability, repetitive elements, gene expression, and key carcinogenic pathways. The previously observed hypermethylation phenotype in HPV-positive (HPV+) tumors compared with HPV-negative tumors was robustly present in the immune-strong (IMU) HPV+ subtype but absent in the highly keratinized (KRT) HPV+ subtype. Methylation levels of IMU tumors were significantly higher in repetitive elements, and methylation showed a significant correlation with genomic stability, consistent with the IMU subtype having more genomic stability and better prognosis. Expression quantitative trait methylation (cis-eQTM) analysis revealed extensive functionally-relevant differences, and differential methylation pathway analysis recapitulated gene expression pathway differences between subtypes. Consistent with their characteristics, KRT and HPV-negative tumors had high regulatory potential for multiple regulators of keratinocyte differentiation, which positively correlated with an expression-based keratinization score. Together, our findings revealed distinct mechanisms of carcinogenesis between subtypes in HPV+ HNSCC and uncovered previously ignored epigenomic differences and clinical implications, illustrating the importance of fine-scale subtype analysis in cancer.

**Significance:**

This study revealed that the previously observed hypermethylation of HPV(+) HNSCC is due solely to the IMU subtype, illustrating the importance of fine-scale subtype analysis in such a heterogeneous disease. Particularly, IMU has significantly higher methylation of transposable elements, which can be tested as a prognosis biomarker in future translational studies.

## Introduction

DNA methylation regulates gene expression by controlling transcription factor (TF) binding, silencing tumor suppressor genes or activating oncogenes, which contributes to carcinogenesis. Both global and local aberrant methylation patterns are associated with cancer survival (1), and methylation signatures have been proposed as biomarkers for both prognosis and treatment response (2). Hypermethylation of promoter-associated CpG islands in cancer is well recognized as a mechanism to silence tumor suppressor genes (1). Conversely, extensive global hypomethylation, especially in repetitive elements, induces genomic instability by enhancing chromosomal rearrangement, leading to tumorigenesis (1, 3, 4). These changes often occur early in the carcinogenic process and are often observed in adjacent “normal” tissue in addition to the tumor, which led to the concept of a methylation field effect (5). Because of its early occurrence, both global methylation levels of a tumor and its unique local patterns of methylation can indicate distinct oncogenic phenotypes, such as stronger relative reliance on chromosomal aberrations, epithelial-to-mesenchymal transition (EMT), or host immune suppression.

DNA methylation has also been useful in defining and characterizing cancer subtypes, which are important to understand because subtypes often follow different trajectories in prognosis, locoregional spread versus distant metastasis, and treatment response (6). Head and neck squamous cell carcinoma (HNSCC) is a prime example, in which tumor subtypes have critical differences in carcinogenic mechanisms, treatment, and prognosis (7). The two main subtypes of HNSCC are associated with profound tobacco and alcohol use and high-risk human papillomavirus (HPV) infection. These subtypes have been further subdivided with significantly different clinical outcomes (7).

Most HPV-induced HNSCCs [HPV-positive HPV(+)] are oropharyngeal and are associated with better prognosis and response to treatment than HPV-negative [HPV(−)] HNSCCs (8). In a subset of these tumors, HPV oncogenes are integrated into the host genome, which may further drive carcinogenesis by upregulating a key oncogene or via alternative mechanisms (9). Several TFs in HNSCC are differentially regulated on the basis of HPV status. The proinflammatory NFκB is variably activated in HPV(+) versus HPV(−) HNSCC, which may be due to differences between canonical and noncanonical forms (10). HPV proteins inhibit NFκB; however, the noncanonical pathway is overexpressed in HPV(+) tumors, with higher activity associated with longer survival (11). In oral cancers more generally, NFκB has been described as having both protumor and antitumor activity, serving as a potential target for the treatment of HNSCC (11, 12). Activator protein-1 (AP-1) TFs, including JunB, are master regulators of epithelial differentiation, and also differ by HPV status, with higher expression in HPV(−) tumors (13), reflecting the fact that HPV(+) HNSCCs are generally less differentiated than HPV(−) (14). HPV-associated changes in the activity of these TFs likely contribute to the distinct transcriptional profiles and clinical outcome differences observed in HPV(+) versus HPV(−) HNSCC.

HPV(+) HNSCC is widely reported to be hypermethylated compared with HPV(−) HNSCC (15, 16), owing to well-studied molecular mechanisms. HPV oncoproteins E6 and E7 activate two separate pathways that promote DNA methyltransferase 1 (DNMT1) expression: (i) E6 inhibition of p53 leads to repression of the specificity protein 1 (SP1)-p53-dependent inhibition of DNMT1 expression and (ii) E7 downregulates the retinoblastoma protein (pRb), leading to re-expression of both E2F and DNMT1 (17). In addition to HPV-induced global methylation changes, studies investigating methylation patterns in HPV(+) HNSCC have demonstrated that certain genes are more likely to have hypermethylated sites (18). These differentially methylated genes are involved in cell-cycle regulation, apoptosis, cellular adhesion, migration, and differentiation (17, 19). For example, hypermethylation of *CDKN2A, RASSF1,* and *CCNA1*, involved in cell cycle and apoptosis, has been outlined in several articles (15, 17, 19). As a major class of molecules regulating cellular adhesion, cadherin family proteins have also been identified with hypermethylated promoter regions in HPV-driven cervical cancer and HNSCC (20, 21).

Until recently, heterogeneity within HPV(+) HNSCCs was not well recognized. Patients with HPV(+) oropharyngeal cancer are generally treated uniformly, with decisions based only on tumor stage and potentially smoking status. Currently, two subtypes are recognized: an immune-strong subtype (IMU) and a highly keratinized subtype (KRT; ref. 22), with KRT potentially further divided by high versus low stromal content (23). Heterogeneity within HPV(+) tumors includes DNA methylation, 5-hydroxymethylation, genomic mutations, and HPV gene integration into the host genome (24). Recently, we identified distinct DNA methylation tumor clusters with hypermethylation enriched in IMU/cervical-like/HPV integration-negative/immune “hot” subtypes (24). Here, we examined DNA methylation profiles using whole-genome bisulfite sequencing (WGBS) in 36 deeply characterized oral tumors, representing the two main subtypes [18 HPV(+) and 18 HPV(−)] as well as a near balance of the HPV(+) fine-grained subtypes (8 IMU and 10 KRT; refs. 22, 25–27). The addition of WGBS data allows insightful connections between previously studied tumor biological characteristics, clinical outcomes, and DNA methylation at distinct genomic regions. More broadly, our results highlight the importance of fine-scale subtype analysis for biomarker discovery, clinical decision making, and clinical trial development.

## Materials and Methods

### WGBS and Data Preprocessing

The recruitment and pathologic review of the 36 patients with HNSCC in this study have been described previously (22, 25, 26). All samples were processed for WGBS library preparation at the University of Michigan Epigenomics Core. The WGBS data were aligned against a bisulfite-converted human genome (hg19) using Bowtie2 and Bismark. The loci with C>T and G>A SNPs that confounded the bisulfite conversion and the sex chromosomes that may induce sex-biased methylation due to ChrX imprinting were filtered out. Only CpG sites with moderate coverage ranging from 10x to 500x reads were used for downstream analysis. To increase coverage, we tiled the adjacent CpG sites into 100-bp windows, resulting in an average coverage of 24x across the genome. The samples were clustered and visualized using uniform manifold approximation and projection (UMAP) (see details in Supplementary Materials and Methods).

### Identification of Differentially Methylated Regions

Differentially methylated regions (DMR) between subtypes [IMU vs. KRT, IMU vs. HPV(−), and KRT vs. HPV(−)] and HPV integration status [HPVint(+) vs. HPVint(−)] were identified using the dispersion shrinkage for sequencing (DSS) model implemented in the R *methylSig* package (v0.99.0). CpG regions covered by at least 80% of the samples in each comparative group were interrogated in the analysis, and regions with FDR < 0.05, and absolute methylation difference ≥20% were selected as DMRs. The optimal set of covariates, including sex, age, smoking status (smoker vs. never), stage (IV vs. others), and HPV gene expression levels counts per million (CPM), were selected on the basis of a backward model selection process (see Supplementary Materials and Methods). The identified DMRs were annotated to known genomic regions and compared with randomly generated regions using the *annoratr* R package (v1.18.1).

### Investigation of the Correlation Between DNA Methylation and Gene Expression by cis-eQTM Analysis

To decipher the relationship between DNA methylation and gene expression, we performed cis-eQTM (expression quantitative trait methylation) analysis (28) on the 100-bp CpG tiles and the transcriptomic data of the same cohort (22). Methylation CpG tiles satisfying the following conditions were selected to avoid correlations driven by outliers: covered in at least 80% (*n* = 29) of the samples, their methylation coefficient of variation was greater than the lowest 5th percentile, and the number of fully unmethylated (meth% = 0) samples <33, which we included because some of the regions with highest coefficient of variation were due to only one or two non-zero methylation samples. In total, 3,546,196 CpG tiles were tested, and the correlation between methylation and expression across the 36 samples were evaluated by a linear model, including four covariates (sex, age, stage, and smoking status). The analysis was implemented using the R package *MatrixEQTL* (29) with the parameter “*pvOutputThreshold.cis* = 0.05” to activate the cis-eQTM module. The significant cis-eQTMs were selected by FDR < 0.1 and the distance to a gene's transcription start site (TSS) ≤1 Mb, which is the default setting in *MatrixEQTL*.

### Genomic Instability Calculation

Genomic instability scores for the 36 tumors were calculated using previously published copy-number alteration (CNA) data. The calculation was based on a modified large-scale transition score that summarizes all copy-number changes present across genomic regions larger than 10 Mb. For chromosome arm level regions, we summarized all the CNAs with a fixed value (|copy number detected – 2|), whereas for regions shorter than chromosome arms but larger than 10 Mb with a CNA, we added a binary value (0 for no CNA; 1 for a CNA).

### Gene Set Enrichment Testing

Differential gene expression analysis was performed for IMU versus KRT, IMU versus HPV(−), KRT versus HPV(−), and HPVint(+) versus HPVint(−) using previously published RNA sequencing (RNA-seq) data of the same 36 tumor samples (GSE74927; see details in Supplementary Materials and Methods). GSE testing was performed on the differentially expressed genes using *RNA-enrich* or on the significant DMRs (FDR < 0.05 and absolute change ≥20%) in different genomic regions and in different directions using the *polyenrich* (when the number of DMRs >10,000) or *chipenrich* method (when DMRs < 10,000) in the *chipenrich* Bioconductor package. We utilized the *<5 kb* gene locus definition to represent promoters. Gene Ontology (GO) biological processes, cellular components, and molecular functions were tested, limiting the GO terms annotated to 10–1,500 genes. The top 40 significant GO terms were visualized using *ggplot v2* 3.3.6.

### Overlap of the DMR-associated Genes with Previously Reported Genes Differentially Methylated by HPV status

Genes differentially methylated between HPV(+) and HPV(−) HNSCC were previously summarized in two review articles (17, 19). We summarized the number of DMRs annotated to the promoter and gene body using biomaRt 2.50.3, with the ENSEMBL hg19 gene annotation. The promoters were defined as 1,000 bp regions upstream of TSSs. We also defined the methylation direction of a gene by directly comparing the count of hypermethylated to hypomethylated DMRs detected across the gene body. The average methylation level of each gene was visualized using a modified *plot_gene* function in the Bioconductor packages *NanoMethViz* and *ggplot v2* 3.3.6.

### Comparison with the Methylation Changes Between Tumor and Normal Samples

Methylation levels at CpG sites that were differentially methylated between IMU and KRT in our cohort, and between tumor versus normal samples in The Cancer Genome Atlas (TCGA) HNSC, were clustered and visualized using the *ComplexHeatmap* package (see details in Supplementary Materials and Methods).

### Calculation of Patient-level Methylation and Pathway Scores

The overall methylation levels in particular genomic regions were calculated for individual samples using the *beta_profile_gene_centered.py* and *beta_profile_gene_centered.py* functions of the *CpGtools* Python package. Methylation profiles were compared with 23 relevant normal tissue/cell types from a human DNA methylation atlas dataset (GSE186458; ref. 30). Because the overall patient-level methylation scores could be influenced by the percentages of other cell types present in their tumor samples, we performed two deconvolution analyses to determine the cancer cell-specific methylation levels. Patient-wise expression pathway scores were calculated using RNA-seq data for five representative gene sets (see details in Supplementary Materials and Methods).

### Methylation-based Inference of Regulatory Activity Score Analysis

Using the WGBS data from the 36 patients with HNSCC, Methylation-based Inference of Regulatory Activity (MIRA) scores were calculated using the Bioconductor package *MIRA* to examine the regulatory activity of eight region sets of interest: strong and weak enhancers in normal human epithelial keratinocytes (NHEK) cells and six TFs obtained from the most relevant cell line available (see details in Supplementary Materials and Methods).

### Ethical Compliance

Written informed consents were obtained from the human participants, and the study was conducted in accordance with the U.S. Common Rule. Additionally, the study was approved by the University of Michigan Institutional Review Board.

### Data Availability

WGBS data are available in the Gene Expression Omnibus under accession number GSE180260. The super series of this cohort can be accessed using GSE74956.

## Results

### The Hypermethylated Phenotype of HPV(+) Tumors is Observed Solely in the IMU Subtype

**FIGURE 1:**
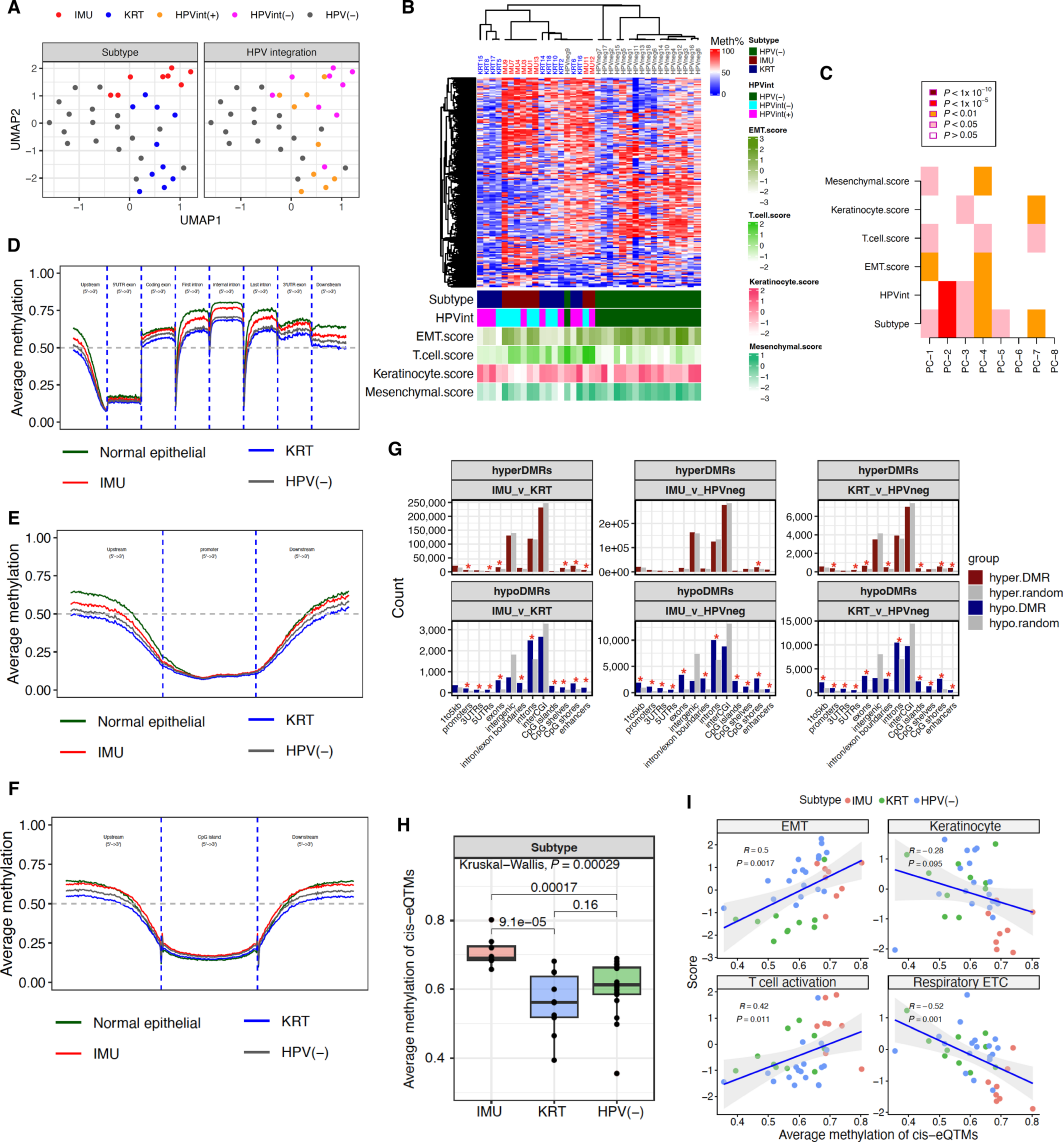
Overview of methylation in the 36 HNSCC samples shows distinct patterns and hypermethylation in the IMU HPV(+) subtype. **A,** UMAP based on methylation levels. **B,** Consensus clustering (*k* = 5) using the top 5,000 variable CpG regions based on median absolute deviation values, along with the heat map visualization. **C,** SVD analysis based on the eight PCs of methylation in the cohort. Average methylation across gene body (**D**), promoter (**E**), and CpG islands (**F**) in normal epithelial cell lines, IMU, KRT subtypes and HPV(−) tumors. **G,** Genomic locations of the identified hyper-DMRs (hypermethylated in first group compared with second; dark red) and hypo-DMRs (hypomethylated in first group compared with second; dark blue) from each comparison. A comparable number of regions were randomly generated (in light grey) and their genomic locations were compared with the DMR locations. The regions significantly enriched in DMRs as compared with random regions are denoted by red asterisks (Fisher exact test, Benjamini–Hochberg adjusted *P* value < 0.05 and OR > 1.5). **H,** Average methylation at cis-eQTM regions grouped by HPV(+) HNSCC subtypes (IMU and KRT) and HPV(−) HNSCC. **I,** Correlation between the average methylation at cis-eQTM regions and HNSCC-related pathway scores, including EMT, keratinocyte differentiation, T-cell activation, and respiratory ETC.

The samples tended to segregate both by HPV status and within the HPV(+) group by IMU and KRT subtypes using dimensionality reduction on the average methylation of 100-bp tiled CpG regions. However, HPV(+) samples did not cluster by HPV integration-positive [HPVint(+)] versus HPV integration-negative [HPVint(−)] status (Fig. 1A). Consensus clustering of the top 5,000 most variable 100 bp CpG regions almost completely separated the HPV(+) and HPV(−) samples, except for sample HPVneg9, which was an outlier in our previous transcriptomic analysis (22). Six of the eight IMU tumors clustered together in a distinct subtree, but no clustering pattern was observed on the basis of HPV integration status (Fig. 1B). As noted previously, IMU demonstrated higher expression of EMT/mesenchymal and T-cell expression scores, and lower keratinocyte differentiation scores (see Materials and Methods) compared with KRT. Singular variable decomposition (SVD) analysis based on the principal components (PC) of methylation supported a strong association with subtype and HPV integration, as well as differentiation pathway scores (Fig. 1C). Thus, although these findings do not robustly recapitulate the transcriptionally defined subtypes, they associate with both the subtypes and relevant carcinogenic pathways.

Next, we investigated the global average methylation profiles of different genomic regions, using the methylation level in normal epithelial cells (31) as the baseline. The genome overall was hypomethylated in KRT and HPV(−) subtypes compared with IMU, most notably in exons and introns, but also around promoters and in regions adjacent to CpG islands (Fig. 1D–F; Supplementary Fig. S1). All tumors exhibited hypomethylation compared with normal epithelial cells in promoters and genic regions. Hypomethylation also existed in HPVint(+) tumors compared with HPVint(−; Supplementary Fig. S2), although to a lesser extent. Nearly all (99%) of the DMRs between IMU and KRT were hypomethylated in KRT (Table 1; Fig. 1F). Tumors of the KRT subtype (*n* = 10/36) were no more hypermethylated than HPV(−) tumors, with more hypomethylated sites (hyper: 34% vs. hypo: 66%). Thus, the hypermethylation phenotype previously ascribed broadly to HPV-associated HNSCC is restricted to the IMU subtype in our cohort. Although DMRs by HPV integration status were nearly all hypermethylated in one group [98% in HPVint(−) tumors], there were less than one-third as many DMRs as by subtype. Thus, given the strong evidence that DNA methylation is driven more by HPV(+) subtype than by HPV integration status, we focus on subtype differences for the remainder of this article.

**TABLE 1:** The total number of tested CpG 100-bp tiles and the number (percentage) of DMRs identified in each comparison

Comparison	Number of analyzed tiles	Number of hyper-DMRs (%)	Number of hypo-DMRs (%)	Total number of DMRs (%)
IMU vs. HPV(−)	3,757,540	305,812 (96%)	14,259 (4%)	320,071 (9%)
IMU vs. KRT	3,383,553	266,762 (99%)	3,564 (1%)	270,326 (8%)
KRT vs. HPV(−)	3,453,805	8,052 (34%)	15,705 (66%)	23,757 (1%)
HPVint(−) vs. HPVint(+)	3,326,189	87,889 (98%)	1,798 (2%)	89,687 (3%)

The hypomethylation in KRT or HPV(−) compared with IMU was not strongly enriched in any genic or CpG island-related regions, consistent with a global undirected mechanism. However, the relatively small number of regions hypermethylated in KRT or HPV(−) compared with IMU were strongly enriched in promoters, exons, and regions in and around CpG islands (Fig. 1G). Very limited overlap was found between regions hypomethylated in KRT and regions previously reported as hyper-hydroxymethylated in KRT compared with IMU (∼0.2% overlap) in the same cohort (27), suggesting that the observed hypomethylation in the KRT subtype was not induced by differences in 5-hydroxymethylation in these tumors.

To investigate the relevance of DNA methylation differences to gene expression, we performed cis-eQTM analysis (see Materials and Methods) and identified 212,850 unique CpG 100-bp tiles that were significantly associated with the expression of 15,031 target genes (FDR < 0.1). Of these, 65,917 (31%) overlapped with a DMR between the three subtypes. Overall, close to half (51%) of the cis-eQTM pairs were negatively associated with gene expression, while for those overlapping a DMR between the subtypes, 47% were negatively associated with gene expression. Although more negative than positive DMR-overlapping cis-eQTMs occurred near CpG islands as expected, a wide spread of both negative and positive cis-eQTMs were observed across genic and CpG island annotations (Supplementary Fig. S3).The DMRs hypomethylated in KRT versus IMU were more enriched in positive cis-eQTMs (9.5% vs. 6.1% negative), whereas the relatively small number of DMRs hypomethylated in IMU versus KRT were enriched in negative cis-eQTMs (12.9% vs. 6.5% positive; Supplementary Table S1), illustrating that both positive and negative regulation of expression is commonly employed by DNA methylation in cancers. Consistent with the overall methylation level, the average methylation at cis-eQTM regions was significantly higher in IMU as compared with KRT or HPV(−) tumors (Fig. 1H) and significantly correlated with key HNSCC carcinogenic pathway scores, including EMT, T-cell activation, keratinization, and respiratory electron transport chain (ETC; Fig. 1I). These findings show that DNA methylation differences across cancer subtypes are associated with important carcinogenic processes.

### Global Methylation is Significantly Anticorrelated with Genomic Instability

**FIGURE 2:**
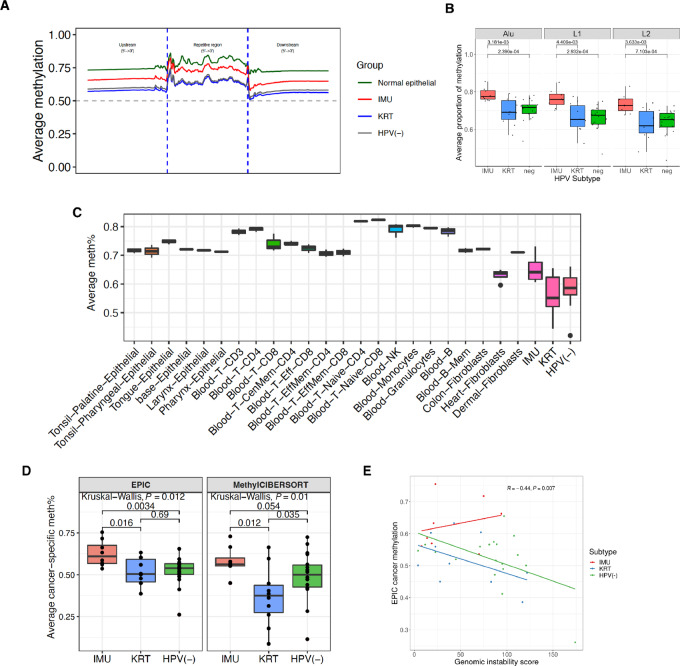
Global methylation is associated with genomic stability and tumor subtype. **A,** Overall methylation profiles at repetitive regions, as well as their upstream and downstream 2 kb regions in normal epithelial cell lines, IMU, KRT subtypes, and HPV(−) tumors. **B,** Average methylation at repetitive elements (Alu, L1, and L2) by subtype. **C,** Boxplots of genome-wide average methylation in normal cell types and subtypes/HPV(−) HNSCC samples of our cohort. **D,** Average cancer-specific methylation grouped by HPV(+) HNSCC subtypes (IMU and KRT) and HPV(−) HNSCC. Cancer-specific methylation levels derived from EPIC and MethylCIBERSORT approaches are shown separately. **E,** Correlation between genomic instability and cancer-specific methylation of the 36 HNSCC samples, denoted by different colors according to their subtypes. The Pearson correlation (*R*) and correlation test *P* value are shown on top right.

Hypomethylation of repetitive elements occurs in many cancer types (32). In particular, hypomethylation of long interspersed nucleotide element-1 (LINE-1) transposable elements is associated with poor prognosis in several cancers and is used as a prognostic biomarker in oropharyngeal cancer (33). This prompted us to investigate the methylation profiles of repetitive elements overall and at the level of individual repetitive element families. Compared with IMU, both KRT and HPV(−) demonstrated lower methylation across all repetitive elements, as well as their upstream and downstream 2 kb regions (Fig. 2A), with significant differences in the Alu, LINE-1, and LINE-2 elements (Fig. 2B). The finding that KRT and HPV(−) are more hypomethylated in repetitive elements is consistent with reports that they have poorer prognosis than IMU subtype patients (22, 33). Moreover, repetitive elements were significantly hypomethylated in advanced HNSCC (stage IV) compared with early-stage HNSCC (stages I–III; Supplementary Fig. S4A).

To interrogate the relationship between overall tumor methylation levels and genomic instability, which has previously been observed in HNSCC (4) as well as other cancers (34–37), we generated patient-wise methylation scores and CNA-based genomic instability scores (see Materials and Methods). First, we compared patient-level methylation in our cohort with normal cell types, which were investigated in a methylation atlas project (31). The average methylation of patients with HNSCC was significantly lower than the comparative normal samples (59% vs. 74%, Wilcoxon rank-sum test, *P* = 6.04 × 10^−14^), with KRT showing the lowest methylation level (Fig. 2C). This is consistent with cancers tending to be globally hypomethylated (1). As tumor tissues are often composed of a mixture of infiltrated immune cells, stromal cells, and tumor cells, overall sample methylation can be confounded with the proportion of non-cancer cells. Thus, to infer the cancer cell–specific methylation levels of individual patients, we utilized the methylation of normal cell lines, including a variety of immune cells and fibroblasts (30), to predict the proportions of normal and tumor cells in our cohort using cell type decomposition analysis (see Materials and Methods). As shown in Fig. 2D, the cancer cell–specific methylation of IMU remained significantly higher than KRT and HPV(−) (Wilcoxon rank-sum test, *P* < 0.05), consistent with the trend of overall unadjusted DNA methylation (Supplementary Fig. S4B and S4C). Cancer cell–specific methylation was significantly anticorrelated with genomic instability (Pearson *R* = −0.44, *P* = 0.007), with IMU showing the highest methylation and lowest genomic instability (methylation mean: 62% [IMU], 51% [KRT], and 52% [HPV(−)]; genomic instability mean: 40.5 [IMU], 54.8 [KRT], and 82.4 [HPV(−)]; Fig. 2E). Interestingly, in contrast to KRT and HPV(−), IMU methylation levels were not negatively correlated with genomic instability, indicating other factors may drive genomic instability in IMU tumors. These findings suggest that hypermethylation in the IMU subtype cannot be explained by their higher levels of immune cell infiltration and the lack of hypomethylation in IMU (in contrast to that observed in KRT) may limit genomic instability during cancer development, leading to a lower chance of HPV integration into the host genome and a better prognostic trajectory compared with KRT and HPV(−) patients.

### Differential Methylation Pathway Analysis Recapitulates Gene Expression Pathway Differences Between Subtypes

**FIGURE 3:**
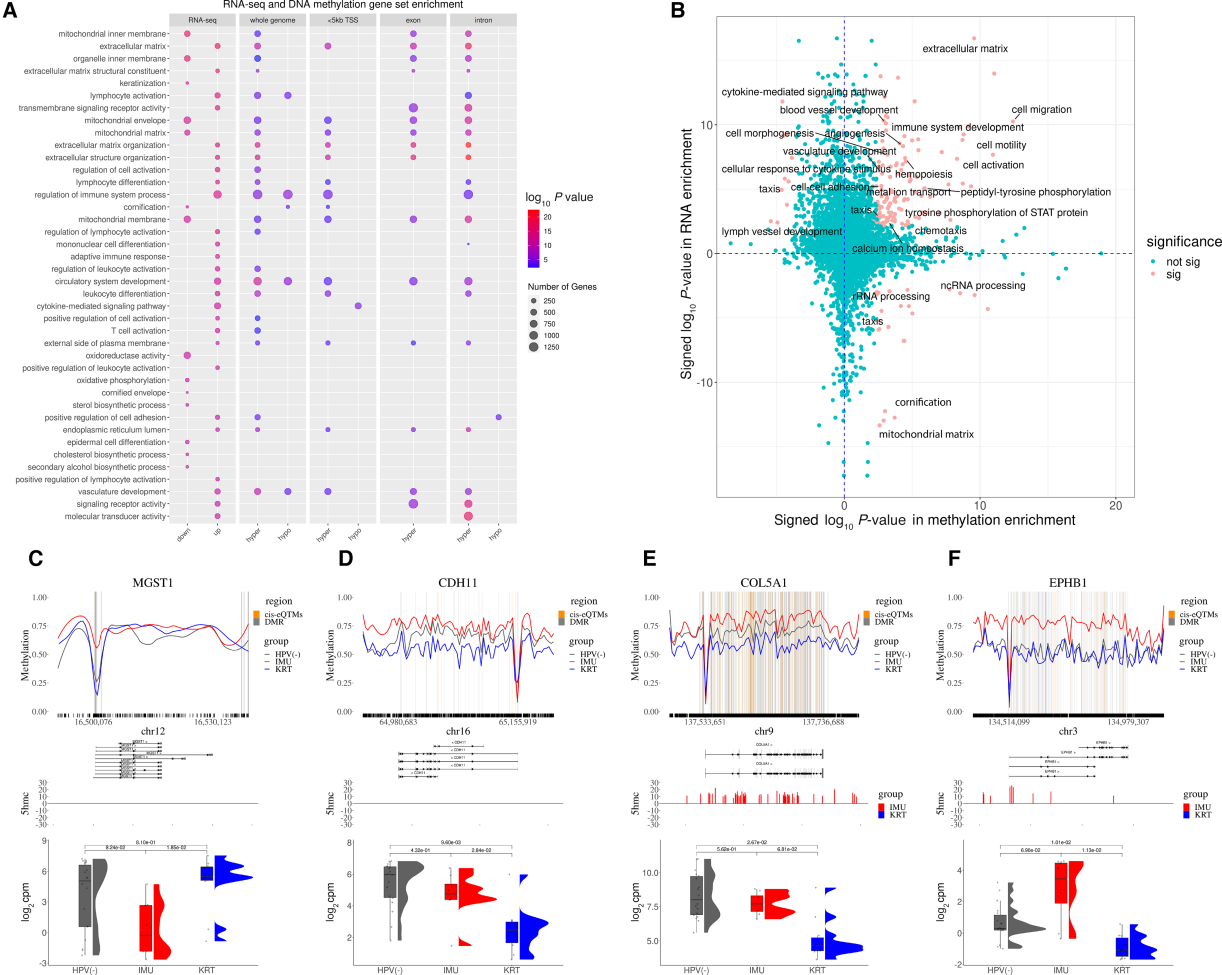
DNA methylation-based gene set enrichment captures gene expression-based pathway-level differences. **A,** Dotplot for top 40 significant RNA enrichment terms, colored by −log_10_(*P*-value) illustrating enrichment results from RNA, DMRs across the whole genome, <5 kb from TSS, exons, and introns. **B,** Scatter plot for directional RNA and DNA methylation enrichment test results. Right quadrants represent significantly hypermethylated GO terms in IMU; top quadrants represent overexpressed terms in IMU. Red: terms significant with FDR < 0.05 for both methylation and expression. **C–F,** Specific genes’ visualization from paradigmatic GO terms, top: DNA methylation profile across gene body with 30% gene length flank regions. Gray lines: IMU versus KRT significant DMRs. Orange lines: Significant cis-eQTMs overlapped with DMRs, middle: differential 5hmc peaks in IMU versus KRT, bottom: bar plot for subgroups’ log_2_cpm expression value with edgeR FDR values. Color: subgroups.

Because most of the DMRs were hypomethylated in KRT, we performed directional enrichment tests separately for DMRs hypermethylated and hypomethylated in IMU versus KRT. The enrichment of GO terms among DMRs between the IMU and KRT subtypes successfully recaptured 29 of the top 40 ranked pathways previously identified between subtypes based on transcriptomic data (22), implying DNA methylation is a crucial epigenetic factor that broadly associates with gene expression differences between HNSCC subtypes (Fig. 3A). Furthermore, we observed that different pathways can be associated with DNA methylation changes in different genomic regions [promoters (5 kb proximal to the TSS), exons, or introns]. For DNA methylation in promoter regions, extracellular matrix and cell adhesion/motility were among the most significantly enriched, with cell differentiation, leukocyte/lymphocyte immune-related processes, and blood vessel morphogenesis among the top-ranked terms (Supplementary Table S2). Oxidative phosphorylation-related processes were not identified via DNA methylation in spite of being highly different in expression between HPV(+) subtypes. This suggests this pathway is regulated via alternative mechanisms, and indeed it is known to be affected by HPV E6 splicing (26).

We next visualized the directional enrichment results for DNA methylation versus gene expression for all GO terms tested, that is we separated enriched hyper-in-IMU versus hyper-in-KRT methylated pathways and up-in-IMU versus up-in-KRT regulated pathways (promoter region methylation results are displayed in Fig. 3B, other regions are shown in Supplementary Fig. S5A and S5B; Supplementary Table S2). For DMRs within promoters, cytokine response terms were only hypermethylated in KRT, not in IMU, with those terms’ genes downregulated in KRT (third quadrant in Fig. 3B). Conversely, terms associated with extracellular matrix and cell adhesion, cell activation and differentiation were hypermethylated in IMU while also being overexpressed in IMU (first quadrant in Fig. 3B). Terms related to rRNA, ncRNA processing, mitochondrial matrix, and cornification (the terminal step in keratinization) display hypermethylation solely in IMU and are overexpressed in KRT (fourth quadrant in Fig. 3B).

Examining genes both differentially expressed and methylated in significant GO terms (see Materials and Methods), we selected four representative genes in top enriched pathways to visualize: Microsomal glutathione-related gene (*MGST1*; Fig. 3C), Cadherin 11 (*CDH11*; Fig. 3D), Collagen V (*COL5A1*; Fig. 3E), and ephrin (*EPHB1*; Fig. 3F), involved in cell adhesion, blood vessel morphogenesis, and tumor suppression. These four genes follow distinct patterns of methylation. *MGST1* has an eQTM in the promoter associating hypermethylation in IMU with downregulation in IMU, while *EPHB1* and *COL5A1* have extensive gene body eQTMs associated with upregulation in IMU [and HPV(−) for *COL5A1*], and *CDH11* displays a combination of these patterns. Furthermore, *COL5A1* and EPHB1 displayed elevated 5-hydroxymethylation in IMU, potentially contributing to increased gene expression in IMU (Fig. 3E and F).

Although “keratinization,” one of the defining features distinguishing these subtypes, was not significant by gene set analysis with DNA methylation, the related terms “cornification” and “epithelial cell differentiation” were enriched (Supplementary Table S2). Examining two key keratinization-related genes (*KRT7* and *KRT6A*), we found that IMU has hypermethylated DMRs in eQTMs at the promoters of *KRT6A* and *KRT7* (Supplementary Fig. S5C and S5D), and both genes were higher expressed in KRT.

### IMU Subtype is the Main Driver of Previously Observed Gene-specific HPV(+) Hypermethylation

**FIGURE 4:**
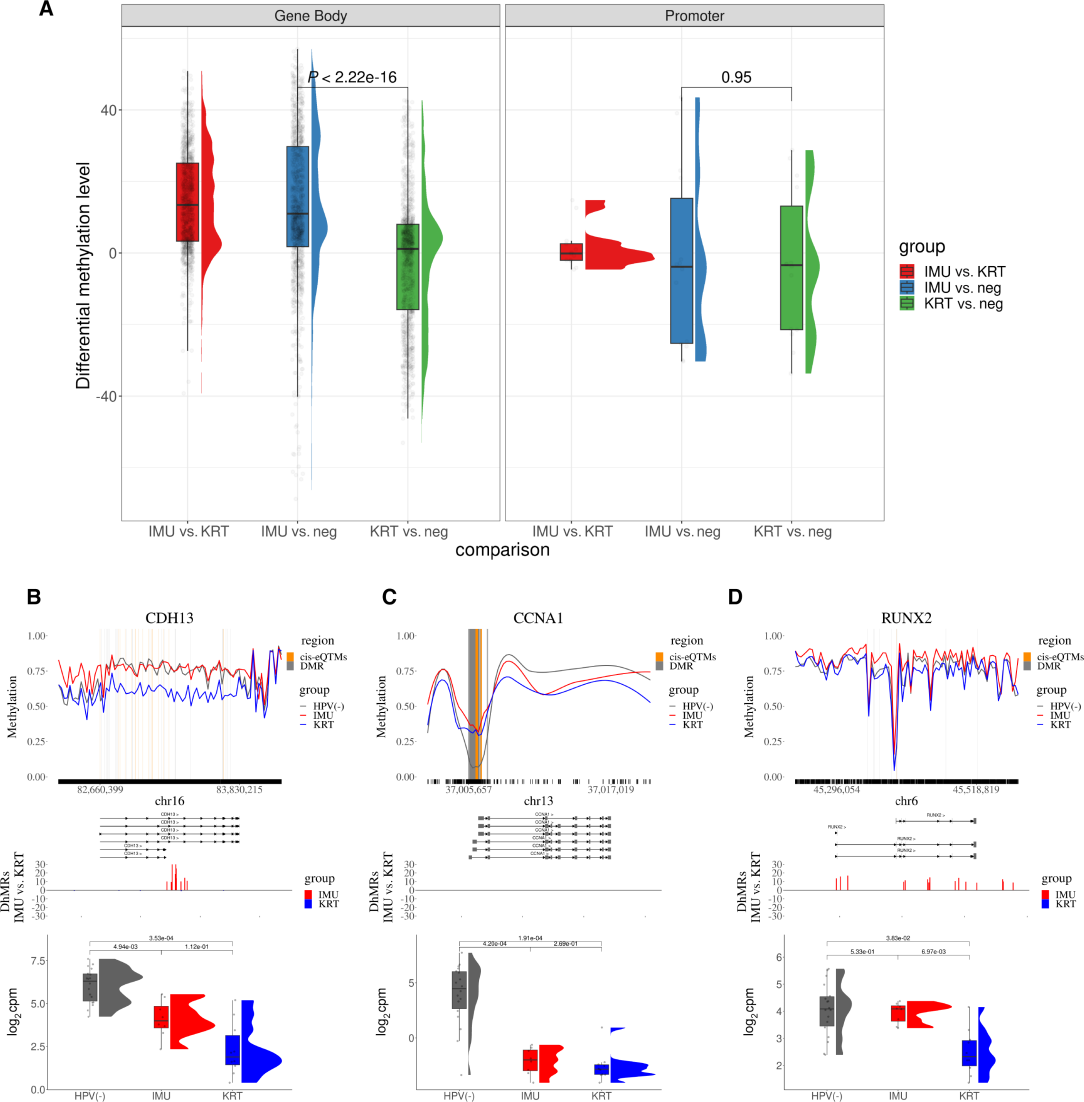
Subtype methylation and expression of genes previously reported as differentially methylated by HPV status. **A,** Rain Cloud plot of differential methylation levels in cis-eQTMs across all 70 genes previously found hypermethylated in HPV(+). **B–D,** Visualization of example genes related to cell adhesion, cell cycle, and differentiation. Figure format is similar to Fig. 3C–F. Gray lines: IMU or KRT versus HPV(−) significant DMRs. Orange lines: significant cis-eQTMs overlapped with gray lines’ DMRs. CCNA1 is an exception where both IMU and KRT HPV+ tumors display hypermethylation compared with HPV(−).

To study the extent to which HPV(+) tumor gene hypermethylation was consistent between subtypes, we examined 108 genes (17, 19) previously reported as differentially methylated in HPV(+) compared with HPV(−) HNSCC (hypo: 37, hyper: 70; Table 2; Supplementary Table S3). Focusing on the 70 hypermethylated genes, we first identified significant cis-eQTMs that overlap with DMRs for IMU versus HPV(−) or KRT versus HPV(−) in specific genomic regions. For these genes’ cis-eQTMs (gene body: 3,370, promoters: 41), IMU exhibited a clear hypermethylation trend compared with HPV(−) in gene bodies, whereas KRT versus HPV(−) methylation differences were centered near zero. In promoter regions with cis-eQTMs, there was no evidence for hypermethylation in either HPV(+) subtype versus HPV(−) (Fig. 4A).

**TABLE 2:** Comparison of promoter and gene body DMRs associated with genes previously reported as differentially methylated by HPV status

Methylation in literature		# DMRs at promoters & direction in our cohort			# DMRs across gene & direction in our cohort		
Gene	HPV(+) vs. HPV(−)	IMU vs. HPV(−)	KRT vs. HPV(−)	IMU vs. KRT	IMU vs. HPV(−)	KRT vs. HPV−	IMU vs. KRT
*CDH13*	hyper	1 hyper	1 hypo	—	16 hyper	233 hypo	177 hyper
					7 hypo		
*CDH23*	hyper	10 hyper	—	5 hyper	558 hyper	2 hypo	445 hyper
					3 hypo		
*ITGA4*	hyper	3 hyper	—	—	7 hyper	—	6 hyper
*RUNX2*	hyper	—	—	—	32 hyper	1 hyper	29 hyper
						2 hypo	
*CCNA1*	hyper	8 hyper	9 hyper	—	8 hyper	8 hyper	—
*CADM1*	hyper	2 hyper	—	1 hyper	22 hyper	—	2 hyper
*CDKN2A*	hypo	—	—	—	2 hyper	2 hypo	6 hyper
*SMC1B*	hypo	—	—	—	11 hyper	3 hyper	—
					7 hypo	6 hypo	
*EPB41L3*	hyper	1 hyper	—	1 hypo	27 hyper	—	2 hyper
							3 hypo
*RUNX3*	hypo	—	1 hypo	2 hyper	13 hyper	4 hypo	90 hyper
					9 hypo		1 hypo
*CHFR*	hypo	1 hyper	—	1 hyper	6 hyper	—	3 hyper
		1 hypo			1 hypo		

Next, we visualized a subset of the 108 genes that were mentioned multiple times in the literature and that represent pathways previously reported as differing by HPV status (cell adhesion, cell cycle and apoptosis, tumor carcinogenesis and progression; Fig. 4B–D; Supplementary Fig. S6). These plots illustrate that *CCNA1* is an exception to the trend, in that it is hypermethylated in both IMU and KRT. They also show the diverse associations between DNA methylation and expression, which depend on pathway and promoter versus gene body methylation.

To further assess differential methylation previously ascribed to HPV(+) versus HPV(−), we defined the differential methylation direction for each of the 108 genes by counting the number of significantly hypermethylated or hypomethylated regions across the gene body (see Materials and Methods). Excluding genes whose previously reported methylation changes conflicted with both our IMU and KRT versus HPV(−) directions, 89/108 (82%) genes were retained [58 of 70 hypermethylated and 31 of 37 hypomethylated in HPV(+)]. Inspecting the 58 hypermethylated genes, only 15 (26%) were hypermethylated in both IMU and KRT versus HPV(−), whereas 43 (74%) were hypermethylated only in IMU versus HPV(−) (Supplementary Table S3). We also examined all 108 gene promoter DMRs (Table 2; Supplementary Table S3). Of the 47 with at least one hypermethylated site in its promoter, 31 (66%) were hypermethylated only in IMU, whereas only four (9%) were hypermethylated solely in KRT versus HPV(−) (Supplementary Table S3). Furthermore, 11 of the 12 remaining genes had fewer hypermethylated regions in KRT than in IMU. This indicates that a large percentage of gene-specific hypermethylation previously found in HPV(+) is likely due to the IMU subtype.

### IMU Displays Hypermethylation in Regions Hypermethylated in Tumor Versus Normal

**FIGURE 5:**
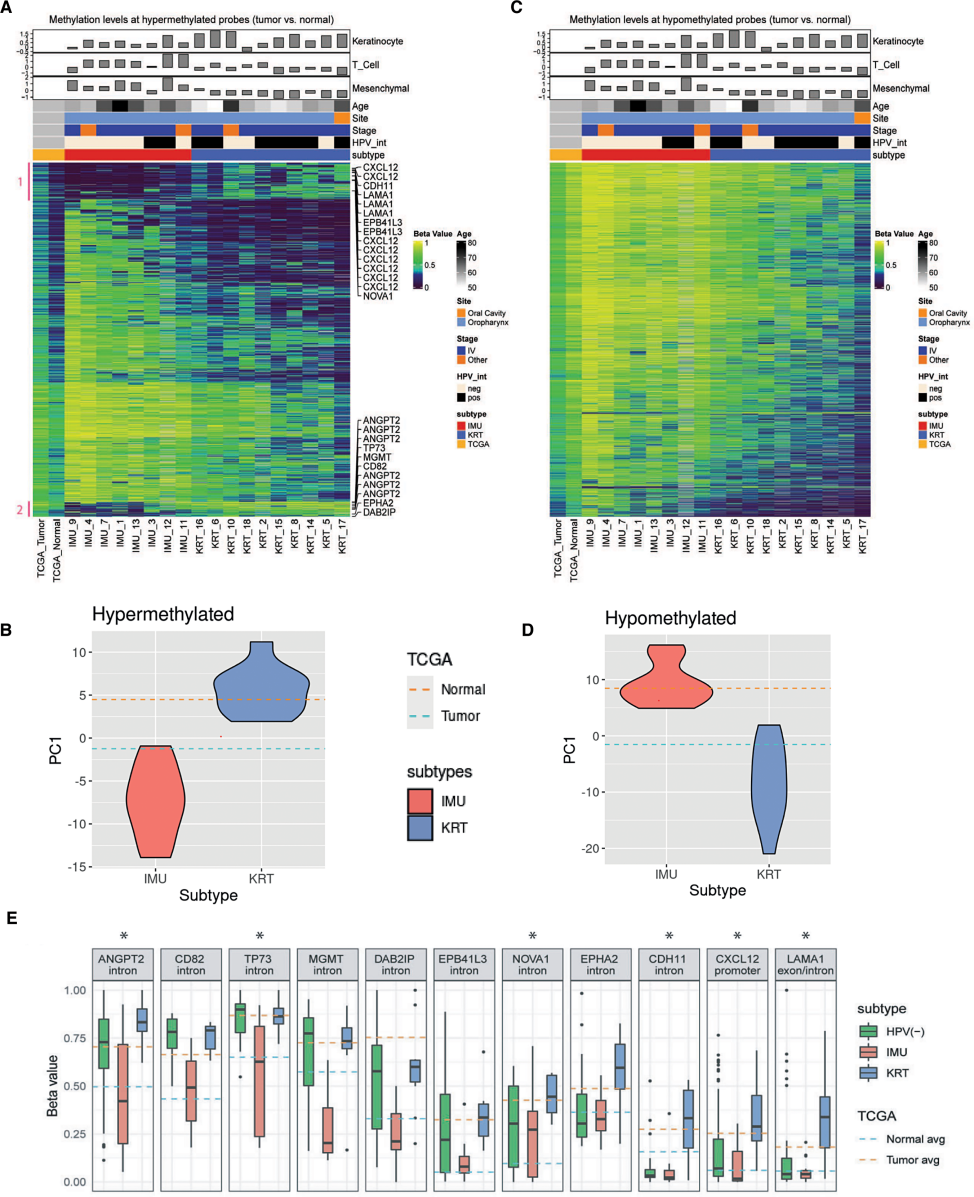
The IMU subtype displays hypermethylation in regions hypermethylated in tumor versus normal; KRT displays hypomethylation in regions hypomethylated in tumor versus normal. Heat maps show the IMU versus KRT DMRs that are hypomethylated (**A**) or hypermethylated (**B**) in TCGA HNSC samples compared to TCGA normal samples. Metadata for our patients is displayed above. **C,** PC analysis of regions hypermethylated in TCGA tumor versus normal for IMU and KRT subtypes. Dashed lines represent average of normal or tumor samples from TCGA. **D,** PC analysis of regions hypomethylated in TCGA tumor versus normal for IMU and KRT subtypes. Dashed lines represent average of normal or tumor samples from TCGA. **E,** Methylation levels at the 100-bp sites marked with the gene symbols of the 11 neoplasm-associated genes found in subclusters 1 and 2 (Fig. 5B). The asterisk indicates significantly repressed expression of that gene in the KRT subtype. In the case multiple sites mapped to a single gene, methylation levels were aggregated and the genomic annotation that appeared most often was used as the annotation in this panel. Note that the HPV(+) methylation levels come from our WGBS data, while TCGA data show methylation levels measured by a set of probes from the Infinium HumanMethylation450 BeadChip by Illumina.

After comparing methylation differences among tumor subtypes, we investigated the methylation patterns displayed by IMU and KRT in regions differentially methylated between HNSCC and normal samples. For this, we utilized TCGA HNSC methylation data, which was measured using Illumina's Infinium HumanMethylation450K BeadChip. Using the 528 tumor and 50 matched normal samples, we identified 91,213 differential CpG sites (FDR < 0.05; 42% hypermethylated and 58% hypomethylated). Comparing the methylation levels among the 18 HPV(+) samples at sites where TCGA HNSC samples were hypomethylated compared with normal, the IMU samples were still hypermethylated and shared a profile more similar to TCGA normal methylation profile, as confirmed by their first PCs from a PCA of their methylation levels (Fig. 5A and B). However, in regions where TCGA HNSC samples were hypermethylated compared with normal, we observed that IMU remained hypermethylated while KRT was more similar to TCGA normal methylation profile (Fig. 5C and D). These results suggest that IMU and KRT follow two distinct carcinogenic pathways via DNA methylation: (i) IMU tends to be hypermethylated at sites commonly hypermethylated in cancers; (ii) in contrast, KRT tends to be hypomethylated globally and in regions that are often hypomethylated in cancer. However, two small subclusters of CpG sites displaying a reversed methylation pattern with KRT being hypermethylated compared with TCGA tumors were of particular interest because they went against the global trend (Fig. 5E, see details in Supplementary Data). Of the 11 neoplasm-related genes in these subclusters, the expression of six (*ANGPT2*, *TP73*, *NOVA1*, *CDH11*, *CXCL12*, and *LAMA1*) were significantly repressed in KRT and one (*EPHA2*) was higher expressed in KRT compared with IMU (Supplementary Fig. S7). Four of these genes, *CXCL12*, *CDH11*, *EPHA2*, and *LAMA1*, are known to be involved in cell adhesion; three of them, *EPHA2*, *ANGPT2*, and *LAMA1*, are involved in PI3K-Akt signaling; and two of them, *CXCL12* and *ANGPT2*, are involved in radiation response.

### Regulatory Potential of TFs Important for Differentiation, Proliferation, and Inflammation Differ by Subtype

**FIGURE 6:**
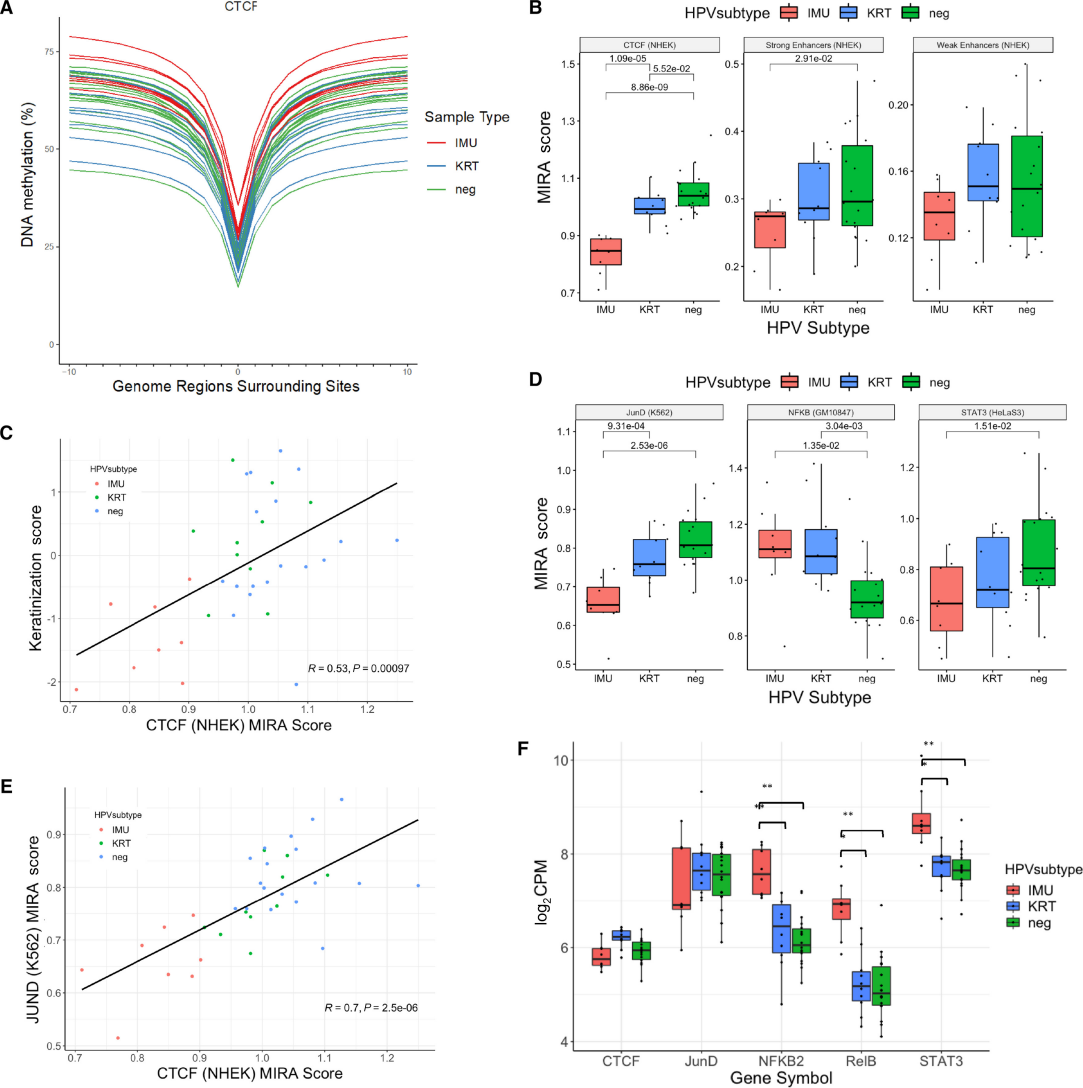
Inferred regulatory activity for genomic regions of interest differ between HNSCC subtypes and correlate with pathway scores. **A,** MIRA profile of CTCF binding sites in the NHEK cell line. Samples are colored by HPV(−), IMU, or KRT. **B,** Box plot displaying MIRA scores separated by subtype for CTCF binding sites, strong enhancers, and weak enhancers each from the NHEK cell line. **C,** Correlation between CTCF MIRA scores and gene expression–based keratinization scores with subtype shown by color. **D,** Box plot of MIRA scores by subtype for JunD, NFκB, and STAT3 binding sites each taken from different cell lines as indicated in the parentheses. **E,** Correlation between CTCF MIRA scores and JunD MIRA scores denoting subtype by color. Both scatter plots display the Pearson correlation coefficient (*R*) and corresponding *P* value. All *P* values < 0.05 are shown and *P* values < 0.00625 are considered significant. **F,** Box plot displaying gene expression values separated by subtype for transcription factors of interest. FDRs < 0.05 are indicated (*, FDR < 0.05; **, FDR < 0.01).

To explore the relationship between tumor subtype and keratinization, the main differentiation pathway in HNSCC, we inferred the regulatory activity of relevant genomic regions. MIRA scores, which are derived from MIRA profiles (Fig. 6A; Supplementary Fig. S8), were calculated for the binding sites of CTCF, a chromatin remodeling factor, and enhancers identified in NHEK (see Materials and Methods; Supplementary Table S4). Both CTCF binding sites and strong enhancers exhibited similar trends among tumors (Pearson *R* = 0.44, *P* = 0.0079; Supplementary Fig. S8E), consistent with CTCF's role in promoting cell type–specific enhancer activity (38). For both CTCF and strong enhancers, IMU had lower inferred regulatory activity than KRT and HPV(−) (Fig. 6B), suggesting IMU is less keratinized than KRT and HPV(−), partly due to a shift in CTCF binding and differential enhancer activity. CTCF MIRA scores were positively correlated with expression-based keratinization (Pearson *R* = 0.53, *P* = 9.7 × 10^−4^; Fig. 6C), consistent with higher keratinization in KRT and HPV(−). This indicates that methylation-based CTCF-inferred binding activity is a biomarker of the degree of keratinization.

The regulatory potential of JunB and JunD binding sites was also analyzed because of their role in epithelial differentiation and proliferation. JunB and JunD belong to the AP-1 family of TFs, which are critical for differentiation of basal (stem cell-like) epithelial cells to mature keratinocytes (13). Considering that the relative importance of JunB and JunD in epithelial differentiation is not well known and that mRNA levels are generally a poor indicator of TF activity (39), we used MIRA scores to identify differences in regulatory potential among subtypes. Both JunB and JunD exhibited a similar trend as CTCF, with IMU having significantly lower regulatory activity than KRT and HPV(−) (Fig. 6D; Supplementary Fig. S8F). JunD also had a strong positive correlation with CTCF (Pearson *R* = 0.70, *P* = 2.5 × 10^−6^; Fig. 6E) and gene expression–based keratinization (Pearson *R* = 0.39, *P* = 0.019; Supplementary Fig. S8G). This suggests differences in cell proliferation among tumor subtypes and illustrates that higher keratinization in KRT and HPV(−) can be explained epigenetically from multiple perspectives. CTCF and JunD MIRA scores were also more strongly correlated with gene expression–based keratinization than with their respective gene expression levels (CTCF: Pearson *R* = 0.29, *P* = 0.091; JunD: Pearson *R* = 0.27, *P* = 0.11), and CTCF and JunD were not differentially expressed between tumor subtypes (Fig. 6F), illustrating how MIRA scores provide regulatory information that is not captured by gene expression levels alone. The regulatory trends of two additional TFs involved in cell proliferation or cellular growth/survival, EZH2 and STAT3, were similar to those of CTCF, JunB, and JunD (Fig. 6D; Supplementary Fig. 8E, see details in Supplementary Data).

Conversely, the regulatory potential of NFκB, a TF involved in regulation of inflammatory and immune responses and implicated in progression of HNSCC, was higher in both IMU and KRT than in HPV(−) (Fig. 6D). NFκB has been reported to be overexpressed in HPV(+) compared with HPV(−) HNSCC. Consistently, our results suggested increased noncanonical NFκB activity in HPV(+) versus HPV(−), with overexpression of the pathway genes RELB and NFKB2 in HPV(+), and even more so in IMU than in KRT (Fig. 6F). However, the MIRA scores for NFκB did not significantly differ between HPV(+) subtypes (Fig. 6D), indicating that other regulatory factors may exist. Taken together, these findings further demonstrate distinct carcinogenic mechanisms in fine-scale subtypes of HNSCC.

## Discussion

DNA methylation, especially in repetitive elements, is one of many molecular correlates of patient prognosis across cancers, with other molecular correlates including signatures of tumor-infiltrating lymphocytes (40), degree of EMT, differentiation status (41), perineural invasion (42), and cancer-associated fibroblast content (43). However, the driving elements of these variables are usually unknown. DNA methylation is an early epigenetic change in several tumor types including HPV-associated HNSCC (44, 45), and also occurs in proximal tissue, indicating a methylation “field effect” and suggesting that it is upstream and potentially causative of later carcinogenic changes (46).

To profile the shift of DNA methylation in tumor tissue in a large cohort of patients, researchers have generally chosen microarray technologies (i.e., Illumina HumanMethylation450 or EPIC BeadChip) with limited genome coverage (less than 5% of CpG sites in the genome and enriched in CpG islands), resulting in scant methylation profiling in gene bodies and distal regulatory elements that play important roles in differentiation. WGBS, which covers >95% of human CpG sites, is regarded as the current “gold standard” technique, enabling a comprehensive map of cancer DNA methylomes, although its cost is often prohibitive. To the best of our knowledge, this is the first study of DNA methylation using WGBS in HPV(+) and HPV(−) HNSCC.

Although a hypermethylated subtype of HPV(+) HNSCC has been previously reported, it has not been well characterized (47) or interpreted in the context of other tumor-defining features. A recent review by Nakagawa and colleagues (19) reported that HPV(+) HNSCCs with high DNA methylation in promoter regions tend to be negative for HPV integration events, may be more likely to have CREB binding protein (*CREBBP*) mutations [found in one of two studies (47, 48)], and have better prognosis. Those with lower promoter methylation correlated with HPV integration and worse prognosis; however, this study used the 450 K BeadChip (47). Although these characteristics suggest a possible association with IMU/KRT subtypes, neither the subtypes nor intergenic and repetitive element methylation were reviewed. In a recent review of HPV(+) subtypes, we noted that the immune-strong/HPVint(−) subtype displayed stronger hypermethylation in regions assayed by the Illumina 450K BeadChip than KRT; however, they were not analyzed relative to HPV(−) or previous findings by HPV status, and analysis was limited by the assay (24). The only WGBS study of HPV(+) tumors was performed for cervical tumors (49), where it was found that, compared with normal and cervical intraepithelial neoplasia, cervical cancer displayed global hypomethylation accompanied by local hypermethylation, consistent with our observation of overall hypomethylation in KRT, which is associated with worse prognosis.

Although our study was limited by sample sizes, especially for the HPV(+) subtypes, one of our striking findings is that global and most of the specific hypermethylated regions attributed to HPV(+) status are found solely in IMU. We observed that IMU appears more similar to normal in regions hypomethylated in cancer, whereas KRT is more similar to normal in regions hypermethylated in cancer. This suggests that each subtype takes advantage of its own key characteristic, that is, its ability to hypermethylate or hypomethylate genomic regions in IMU and KRT, respectively, to promote its own line of carcinogenesis. We also identified several specific sites and genes that are differentially methylated between subtypes and correlated with gene expression as determined by cis-eQTM analysis. However, due to the relatively small sample sizes, these findings should be validated with a larger cohort.

In addition, we found that NFκB showed higher regulatory activity in HPV(+) compared with HPV(−), indicating that HPV(+) tends toward an inflammatory response. There have been mixed findings regarding the role of NFκB in HNSCC development depending on the pathway involved. The canonical NFκB was reported to be a potential target in HNSCC tumors (50); however, it was also observed that inducing the noncanonical NFκB pathway, which was upregulated in HPV(+) tumors, resulted in more IL12-producing dendritic cells and sensitized tumors to anti-PD-1 therapy (51). Therefore, DNA methylation may be part of a predictive biomarker for immunotherapy responses by estimating noncanonical NFκB activity (51). Future research will need to be performed to understand whether and how DNA methylation can be used as a prognostic biomarker or biomarker for immune checkpoint inhibitor (e.g., anti-PD-L1) response.

In HNSCC, HPV(+) status is positively associated with LINE-1 methylation (52). The hypomethylation of transposons, such as LINE-1, promotes genomic structural alterations during cell division and unwanted activation of transposable elements in cancer genomes, leading to somatic mutations (53). Here, we showed that IMU has higher methylation in repetitive element families (including LINE1/2) than in either KRT or HPV(−). This justifies future translational studies using repetitive element DNA methylation markers for de-escalation or escalation treatment trials in patients with HPV-associated oropharyngeal cancer. Lavasanifar and colleagues concluded that the use of LINE-1 DNA methylation as a biomarker for prognosis in several cancer types holds great promise (54), there is currently a lack of studies to validate its use in specific cancer types. Highly methylated tumors tend to be less aggressive with better prognosis, consistent with the better survival observed in IMU patients. Examples include aggressive pancreatic ductal adenocarcinoma, which displays hypomethylation of repetitive elements (55), and LINE-1 hypomethylation, which is strongly associated with poor prognosis in multiple gastrointestinal cancers (56).

Although Nakagawa and colleagues suggested that hypermethylation in HPV(+) HNSCC correlates with improved survival, methylation profiles were not investigated with respect to HPV(+) subtypes. Here, we showed that DNA methylation profiles are significantly associated with the main differentiating characteristics of HPV(+) tumors, that is, subtype, infiltrating immune cells, keratinization level, and EMT, which are known prognostic features (22, 57). Our study shows not only that a high level of DNA methylation heterogeneity exists among HPV(+) HNSCC tumors, but also provides evidence that HPV(+) HNSCC tumors may follow two distinct carcinogenic routes: one characterized by high DNA methylation, high immune infiltration, lack of cell-cell adhesion, and higher EMT (22), and the other characterized by strong hypomethylation, high keratinization, higher genomic instability, and more likely HPV gene integration in the host genome. Our results highlight the importance of HPV(+) subtype in the molecular signatures and clinical care of patients with HNSCC. Because changes in DNA methylation often occur early in carcinogenesis and can be detected in liquid biopsies, they are good biomarker candidates for early diagnosis and prognosis.

## Supplementary Material

Supplementary Table S1 Supplemental Table S1- Summary of differentially methylated regions

Supplementary Table S2 RNA and DNA methylation Gene Set Enrichment results

Supplementary Table S3 Gene specific methylation results

Supplementary Table S4 Supplementary Table S4- TFs and their ChIP-seq peak sets used

Supplementary Results Supplementary Results

Supplementary Methods Supplementary Methods

Supplementary Fig 1 Companion figure showing significance results for main Figure 1D-F and Figure 2A.

Supplementary Fig 2 Average methylation of tumors and normal epithelial cells at gene body (A), promoter (B), CpG island (C) and repetitive region (D). Samples were grouped by HPVor HPV integration status.

Supplementary Fig 3 Comparison of locations of positive versus negative cis-eQTMs that overlap with DMRs for: IMU vs HPV(-) (1st row), IMU vs KRT (2nd row), and KRT vs HPV(-) (3rd row) of each panel. (A) CpG island annotations; (B) genic annotations. 1st and 2nd columns are hypermethylated in the first comparison group; 3rd and 4th columns are hypomethylated in the first comparison group. Negative eQTMs are in the 1st and 3rd columns, while positive eQTMs are in the 2nd and 4th columns.

Supplementary Fig 4 Patient-level methylation at repetitive regions, stratified by disease stage (A), by subtype and HPV status across genome (B) or at different genomic regions (C).

Supplementary Fig 5 Correlate GSE testing results from mRNA expression and DNA methylation in the exon, intron regions and keratinization genes visualization.

Supplementary Fig 6 Methylation and expression visualization of the genes selected from the 108 genes previously reported as differentially methylated in HPV(+) compared to HPV(-) HNSCC.

Supplementary Fig 7 Methylation and expression profiles of the eleven genes that are hypermethylated in TCGA HNSC tumor vs normal, and hypermethylated in KRT vs IMU shown in Figure 5C.

Supplementary Fig 8 (A-D) MIRA profiles of strong and weak enhancers in the NHEK cell line (A), JunD binding sites in the K562 cell line (B), NFKB binding sites in the GM10847 cell line, and STAT3 binding sites in the HeLaS3 cell line (D) Samples are colored by HPV tumor subtype. (E) Correlations between CTCF MIRA scores and Strong Enhancer MIRA scores. (F) Box plot of MIRA scores separated by subtype for EZH2 binding sites and JunB binding sites from NHEK and K562 cell lines, respectively. (G) Correlations between JunD MIRA scores and gene expression-based keratinization scores. All scatter plots separate subtype by color and display the Pearson correlation coefficient (R) along with the corresponding p-value.

## References

[1] Ehrlich M . DNA hypomethylation in cancer cells. Epigenomics 2009;1:239–59. 20495664 10.2217/epi.09.33 PMC2873040

[2] Koch A , Joosten SC, Feng Z, de Ruijter TC, Draht MX, Melotte V, . Analysis of DNA methylation in cancer: location revisited. Nat Rev Clin Oncol 2018;15:459–66. 29666440 10.1038/s41571-018-0004-4

[3] Ross JP , Rand KN, Molloy PL. Hypomethylation of repeated DNA sequences in cancer. Epigenomics 2010;2:245–69. 22121873 10.2217/epi.10.2

[4] Richards KL , Zhang B, Baggerly KA, Colella S, Lang JC, Schuller DE, . Genome-wide hypomethylation in head and neck cancer is more pronounced in HPV-negative tumors and is associated with genomic instability. PLoS One 2009;4:e4941. 19293934 10.1371/journal.pone.0004941 PMC2654169

[5] Luo Y , Yu M, Grady WM. Field cancerization in the colon: a role for aberrant DNA methylation? Gastroenterol Rep 2014;2:16–20. 10.1093/gastro/got039 PMC3920999 24760232

[6] Heyn H , Esteller M. DNA methylation profiling in the clinic: applications and challenges. Nat Rev Genet 2012;13:679–92. 22945394 10.1038/nrg3270

[7] De Cecco L , Nicolau M, Giannoccaro M, Daidone MG, Bossi P, Locati L, . Head and neck cancer subtypes with biological and clinical relevance: meta-analysis of gene-expression data. Oncotarget 2015;6:9627–42. 25821127 10.18632/oncotarget.3301 PMC4496244

[8] Perri F , Longo F, Caponigro F, Sandomenico F, Guida A, Vittoria Scarpati GD, . Management of HPV-related squamous cell carcinoma of the head and neck: pitfalls and caveat. Cancers 2020;12:975. 32326465 10.3390/cancers12040975 PMC7226389

[9] Symer DE , Akagi K, Geiger HM, Song Y, Li G, Emde A-K, . Diverse tumorigenic consequences of human papillomavirus integration in primary oropharyngeal cancers. Genome Res 2022;32:55–70. 34903527 10.1101/gr.275911.121 PMC8744672

[10] Verma G , Vishnoi K, Tyagi A, Jadli M, Singh T, Goel A, . Characterization of key transcription factors as molecular signatures of HPV-positive and HPV-negative oral cancers. Cancer Med 2017;6:591–604. 28155253 10.1002/cam4.983 PMC5345654

[11] Schrank TP , Prince AC, Sathe T, Wang X, Liu X, Alzhanov DT, . NF-κB over-activation portends improved outcomes in HPV-associated head and neck cancer. Oncotarget 2022;13:707–22. 35634245 10.18632/oncotarget.28232 PMC9131933

[12] Vander Broek R , Snow GE, Chen Z, Van Waes C. Chemoprevention of head and neck squamous cell carcinoma through inhibition of NF-κB signaling. Oral Oncol 2014;50:930–41. 24177052 10.1016/j.oraloncology.2013.10.005 PMC4002662

[13] Eckert RL , Adhikary G, Young CA, Jans R, Crish JF, Xu W, . AP1 transcription factors in epidermal differentiation and skin cancer. J Skin Cancer 2013;2013:537028. 23762562 10.1155/2013/537028 PMC3676924

[14] White EA . Manipulation of epithelial differentiation by HPV oncoproteins. Viruses 2019;11:369. 31013597 10.3390/v11040369 PMC6549445

[15] Sartor MA , Dolinoy DC, Jones TR, Colacino JA, Prince MEP, Carey TE, . Genome-wide methylation and expression differences in HPV(+) and HPV(-) squamous cell carcinoma cell lines are consistent with divergent mechanisms of carcinogenesis. Epigenetics 2011;6:777–87. 21613826 10.4161/epi.6.6.16216 PMC3142368

[16] van Kempen PMW , Noorlag R, Braunius WW, Stegeman I, Willems SM, Grolman W. Differences in methylation profiles between HPV-positive and HPV-negative oropharynx squamous cell carcinoma: a systematic review. Epigenetics 2014;9:194–203. 24169583 10.4161/epi.26881 PMC3962529

[17] Ekanayake WC , Tang KD, Vasani S, Langton-Lockton J, Kenny L, Punyadeera C. DNA methylation changes in human papillomavirus-driven head and neck cancers. Cells 2020;9:1359. 32486347 10.3390/cells9061359 PMC7348958

[18] Anayannis NVJ , Schlecht NF, Belbin TJ. Epigenetic mechanisms of human papillomavirus-associated head and neck cancer. Arch Pathol Lab Med 2015;139:1373–8. 25978766 10.5858/arpa.2014-0554-RA

[19] Nakagawa T , Kurokawa T, Mima M, Imamoto S, Mizokami H, Kondo S, . DNA methylation and HPV-associated head and neck cancer. Microorganisms 2021;9:801. 33920277 10.3390/microorganisms9040801 PMC8069883

[20] Lechner M , Fenton T, West J, Wilson G, Feber A, Henderson S, . Identification and functional validation of HPV-mediated hypermethylation in head and neck squamous cell carcinoma. Genome Med 2013;5:15. 23419152 10.1186/gm419 PMC3706778

[21] Wang K-H , Lin C-J, Liu C-J, Liu D-W, Huang R-L, Ding D-C, . Global methylation silencing of clustered proto-cadherin genes in cervical cancer: serving as diagnostic markers comparable to HPV. Cancer Med 2015;4:43–55. 25418975 10.1002/cam4.335 PMC4312117

[22] Zhang Y , Koneva LA, Virani S, Arthur AE, Virani A, Hall PB, . Subtypes of HPV-positive head and neck cancers are associated with HPV characteristics, copy number alterations, PIK3CA mutation, and pathway signatures. Clin Cancer Res 2016;22:4735–45. 27091409 10.1158/1078-0432.CCR-16-0323 PMC5026546

[23] Locati LD , Serafini MS, Iannò MF, Carenzo A, Orlandi E, Resteghin C, . Mining of self-organizing map gene-expression portraits reveals prognostic stratification of HPV-positive head and neck squamous cell carcinoma. Cancers 2019;11:1057. 31357501 10.3390/cancers11081057 PMC6721309

[24] Qin T , Li S, Henry LE, Liu S, Sartor MA. Molecular tumor subtypes of HPV-positive head and neck cancers: biological characteristics and implications for clinical outcomes. Cancers 2021;13:2721. 34072836 10.3390/cancers13112721 PMC8198180

[25] Koneva LA , Zhang Y, Virani S, Hall PB, McHugh JB, Chepeha DB, . HPV integration in HNSCC correlates with survival outcomes, immune response signatures, and candidate drivers. Mol Cancer Res 2018;16:90–102. 28928286 10.1158/1541-7786.MCR-17-0153 PMC5752568

[26] Qin T , Koneva LA, Liu Y, Zhang Y, Arthur AE, Zarins KR, . Significant association between host transcriptome-derived HPV oncogene E6* influence score and carcinogenic pathways, tumor size, and survival in head and neck cancer. Head Neck 2022;42:2375–89. 10.1002/hed.26244 PMC8052131 32406560

[27] Liu S , de Medeiros MC, Fernandez EM, Zarins KR, Cavalcante RG, Qin T, . 5-Hydroxymethylation highlights the heterogeneity in keratinization and cell junctions in head and neck cancers. Clin Epigenetics 2020;12:175. 33203436 10.1186/s13148-020-00965-8 PMC7672859

[28] Hanson C , Cairns J, Wang L, Sinha S. Principled multi-omic analysis reveals gene regulatory mechanisms of phenotype variation. Genome Res 2018;28:1207–16. 29898900 10.1101/gr.227066.117 PMC6071639

[29] Shabalin AA . Matrix eQTL: ultra fast eQTL analysis via large matrix operations. Bioinformatics 2012;28:1353–8. 22492648 10.1093/bioinformatics/bts163 PMC3348564

[30] Loyfer N , Magenheim J, Peretz A, Cann G, Bredno J, Klochendler A, . A human DNA methylation atlas reveals principles of cell type-specific methylation and identifies thousands of cell type-specific regulatory elements. bioRxiv 2022. Available from: https://www.biorxiv.org/content/10.1101/2022.01.24.477547v1.abstract.

[31] Loyfer N , Magenheim J, Peretz A, Cann G, Bredno J, Klochendler A, . A DNA methylation atlas of normal human cell types. Nature 2023;613:355–64. 36599988 10.1038/s41586-022-05580-6 PMC9811898

[32] Pappalardo XG , Barra V. Losing DNA methylation at repetitive elements and breaking bad. Epigenetics Chromatin 2021;14:25. 34082816 10.1186/s13072-021-00400-z PMC8173753

[33] Furlan C , Polesel J, Barzan L, Franchin G, Sulfaro S, Romeo S, . Prognostic significance of LINE-1 hypomethylation in oropharyngeal squamous cell carcinoma. Clin Epigenetics 2017;9:58. 28572862 10.1186/s13148-017-0357-z PMC5450111

[34] Kawano H , Saeki H, Kitao H, Tsuda Y, Otsu H, Ando K, . Chromosomal instability associated with global DNA hypomethylation is associated with the initiation and progression of esophageal squamous cell carcinoma. Ann Surg Oncol 2014;21:S696–702. 24898425 10.1245/s10434-014-3818-z

[35] Marinoni I , Wiederkeher A, Wiedmer T, Pantasis S, Di Domenico A, Frank R, . Hypo-methylation mediates chromosomal instability in pancreatic NET. Endocr Relat Cancer 2017;24:137–46. 28115389 10.1530/ERC-16-0554

[36] Matsuzaki K , Deng G, Tanaka H, Kakar S, Miura S, Kim YS. The relationship between global methylation level, loss of heterozygosity, and microsatellite instability in sporadic colorectal cancer. Clin Cancer Res 2005;11:8564–9. 16361538 10.1158/1078-0432.CCR-05-0859

[37] Rodriguez J , Frigola J, Vendrell E, Risques R-A, Fraga MF, Morales C, . Chromosomal instability correlates with genome-wide DNA demethylation in human primary colorectal cancers. Cancer Res 2006;66:8462–9468. 16951157 10.1158/0008-5472.CAN-06-0293

[38] Ren G , Jin W, Cui K, Rodrigez J, Hu G, Zhang Z, . CTCF-mediated enhancer-promoter interaction is a critical regulator of cell-to-cell variation of gene expression. Mol Cell 2017;67:1049–58. 28938092 10.1016/j.molcel.2017.08.026 PMC5828172

[39] Hernandez JM , Floyd DH, Weilbaecher KN, Green PL, Boris-Lawrie K. Multiple facets of junD gene expression are atypical among AP-1 family members. Oncogene 2008;27:4757–67. 18427548 10.1038/onc.2008.120 PMC2726657

[40] Gooden MJM , de Bock GH, Leffers N, Daemen T, Nijman HW. The prognostic influence of tumour-infiltrating lymphocytes in cancer: a systematic review with meta-analysis. Br J Cancer 2011;105:93–103. 21629244 10.1038/bjc.2011.189 PMC3137407

[41] Ribatti D , Tamma R, Annese T. Epithelial-mesenchymal transition in cancer: a historical overview. Transl Oncol 2020;13:100773. 32334405 10.1016/j.tranon.2020.100773 PMC7182759

[42] Chen S-H , Zhang B-Y, Zhou B, Zhu C-Z, Sun L-Q, Feng Y-J. Perineural invasion of cancer: a complex crosstalk between cells and molecules in the perineural niche. Am J Cancer Res 2019;9:1–21. 30755808 PMC6356921

[43] Paulsson J , Micke P. Prognostic relevance of cancer-associated fibroblasts in human cancer. Semin Cancer Biol 2014;25:61–8. 24560651 10.1016/j.semcancer.2014.02.006

[44] Zhou C , Ye M, Ni S, Li Q, Ye D, Li J, . DNA methylation biomarkers for head and neck squamous cell carcinoma. Epigenetics 2018;13:398–409. 29927694 10.1080/15592294.2018.1465790 PMC6140809

[45] Locke WJ , Guanzon D, Ma C, Liew YJ, Duesing KR, Fung KYC, . DNA methylation cancer biomarkers: translation to the clinic. Front Genet 2019;10:1150. 31803237 10.3389/fgene.2019.01150 PMC6870840

[46] Ramachandran K , Singal R. DNA methylation and field cancerization. Epigenomics 2012;4:243–5. 22690658 10.2217/epi.12.12

[47] Nakagawa T , Matsusaka K, Misawa K, Ota S, Fukuyo M, Rahmutulla B, . Stratification of HPV-associated and HPV-negative oropharyngeal squamous cell carcinomas based on DNA methylation epigenotypes. Int J Cancer 2020;146:2460–74. 31997344 10.1002/ijc.32890

[48] Ando M , Saito Y, Xu G, Bui NQ, Medetgul-Ernar K, Pu M, . Chromatin dysregulation and DNA methylation at transcription start sites associated with transcriptional repression in cancers. Nat Commun 2019;10:2188. 31097695 10.1038/s41467-019-09937-w PMC6522544

[49] Han Y , Ji L, Guan Y, Ma M, Li P, Xue Y, . An epigenomic landscape of cervical intraepithelial neoplasia and cervical cancer using single-base resolution methylome and hydroxymethylome. Clin Transl Med 2021;11:e498. 34323415 10.1002/ctm2.498 PMC8288011

[50] Monisha J , Roy NK, Bordoloi D, Kumar A, Golla R, Kotoky J, . Nuclear factor kappa B: a potential target to persecute head and neck cancer. Curr Drug Targets. 2017;18:232–53. 26844566 10.2174/1389450117666160201112330

[51] Garris CS , Arlauckas SP, Kohler RH, Trefny MP, Garren S, Piot C, . Successful anti-PD-1 cancer immunotherapy requires T cell-dendritic cell crosstalk involving the cytokines IFN-γ and IL-12. Immunity 2018;49:1148–61. 30552023 10.1016/j.immuni.2018.09.024 PMC6301092

[52] Camuzi D , Buexm LA, de Queiroz Chaves Lourenço S, Cuenin C, de Souza Almeida Lopes M, Manara F, . HPV infection leaves a DNA methylation signature in oropharyngeal cancer affecting both coding genes and transposable elements. Cancers 2021;13:3621. 34298834 10.3390/cancers13143621 PMC8306428

[53] Burns KH . Transposable elements in cancer. Nat Rev Cancer 2017;17:415–24. 28642606 10.1038/nrc.2017.35

[54] Lavasanifar A , Sharp CN, Korte EA, Yin T, Hosseinnejad K, Jortani SA. Long interspersed nuclear element-1 mobilization as a target in cancer diagnostics, prognostics and therapeutics. Clin Chim Acta 2019;493:52–62. 30776360 10.1016/j.cca.2019.02.015

[55] Espinet E , Gu Z, Imbusch CD, Giese NA, Büscher M, Safavi M, . Aggressive PDACs show hypomethylation of repetitive elements and the execution of an intrinsic IFN program linked to a ductal cell of origin. Cancer Discov 2021;11:638–59. 33060108 10.1158/2159-8290.CD-20-1202 PMC9216338

[56] Baba Y , Yagi T, Sawayama H, Hiyoshi Y, Ishimoto T, Iwatsuki M, . Long interspersed element-1 methylation level as a prognostic biomarker in gastrointestinal cancers. Digestion 2018;97:26–30. 29393154 10.1159/000484104

[57] Johnson DE , Burtness B, Leemans CR, Lui VWY, Bauman JE, Grandis JR. Head and neck squamous cell carcinoma. Nat Rev Dis Primers 2020;6:92. 33243986 10.1038/s41572-020-00224-3 PMC7944998

