# Supplementary material for: Whole-genome CpG-resolution DNA Methylation Profiling of HNSCC Reveals Distinct Mechanisms of Carcinogenesis for Fine-scale HPV+ Cancer Subtypes
Source: Cancer Res Commun. 2023 Aug 30;3(8):1701–15. doi: 10.1158/2767-9764.CRC-23-0009 (PMC10467604; doi:10.1158/2767-9764.CRC-23-0009)
Supplement: Supplementary Results [file crc-23-0009-s05.docx]

**Supplementary results**

***Regulatory activity of EZH2 and STAT3***

EZH2, a TF involved in cell proliferation, has been associated with poor prognosis in HNSCC [1]. MIRA scores for EZH2 showed a trend similar to that of CTCF, JunB, and JunD, although with no significant differences between the subtypes and high variation in HPV(-) tumors **(Figure 6 A and 6C, Figure S7B)**. Notably, MIRA scores were significantly negatively correlated between EZH2 and JunD (Pearson’s R=-0.43, p=0.0096), and JunB (Pearson’s R=-0.46, p=0.0043). This indicates that regulatory activity of EZH2 and JunB/D in cell differentiation and proliferation was nonreciprocal.

Another TF, STAT3, is involved in cellular growth and survival [2], and its overactivation has been associated with resistance to treatment and immune escape [3]. Within STAT3 binding sites, HPV(-) had the greatest regulatory potential whereas IMU had the lowest **(Figure 6C);** lower STAT3 regulatory activity in IMU may contribute to this subtype’s better prognosis. Notably, the trend in MIRA scores across IMU, KRT and HPV(-) for STAT3 are similar to that of JunB and JunD. As the STAT3 signaling pathway may crosstalk with NF-kB and AP-1 [4,5], the MIRA scores for these TFs may provide insight into cooperative behavior.

**References**

1. Chang, J.W.; Gwak, S.Y.; Shim, G.-A.; Liu, L.; Lim, Y.C.; Kim, J.M.; Jung, M.G.; Koo, B.S. EZH2 Is Associated with Poor Prognosis in Head-and-Neck Squamous Cell Carcinoma via Regulating the Epithelial-to-Mesenchymal Transition and Chemosensitivity. *Oral Oncol.* **2016**, *52*, 66–74.

2. Gaykalova, D.A.; Manola, J.B.; Ozawa, H.; Zizkova, V.; Morton, K.; Bishop, J.A.; Sharma, R.; Zhang, C.; Michailidi, C.; Considine, M.; et al. NF-ΚB and Stat3 Transcription Factor Signatures Differentiate HPV-Positive and HPV-Negative Head and Neck Squamous Cell Carcinoma. *Int. J. Cancer* **2015**, *137*, 1879–1889.

3. Geiger, J.L.; Grandis, J.R.; Bauman, J.E. The STAT3 Pathway as a Therapeutic Target in Head and Neck Cancer: Barriers and Innovations. *Oral Oncol.* **2016**, *56*, 84–92.

4. Verma, G.; Vishnoi, K.; Tyagi, A.; Jadli, M.; Singh, T.; Goel, A.; Sharma, A.; Agarwal, K.; Prasad, S.C.; Pandey, D.; et al. Characterization of Key Transcription Factors as Molecular Signatures of HPV-Positive and HPV-Negative Oral Cancers. *Cancer Med.* **2017**, *6*, doi:10.1002/cam4.983.

5. Grivennikov, S.I.; Karin, M. Dangerous Liaisons: STAT3 and NF-ΚB Collaboration and Crosstalk in Cancer. *Cytokine & Growth Factor Reviews* 2010, *21*, 11–19.
