# Supplementary material for: Whole-genome CpG-resolution DNA Methylation Profiling of HNSCC Reveals Distinct Mechanisms of Carcinogenesis for Fine-scale HPV+ Cancer Subtypes
Source: Cancer Res Commun. 2023 Aug 30;3(8):1701–15. doi: 10.1158/2767-9764.CRC-23-0009 (PMC10467604; doi:10.1158/2767-9764.CRC-23-0009)
Supplement: Supplementary Methods [file crc-23-0009-s06.docx]

**Supplementary Methods**

***Whole genome bisulfite sequencing***

For each sample, the genomic DNA (gDNA) was quantified with the Qubit Broad Range dsDNA kit (Q32856; Invitrogen) and assessed for quality on the TapeStation 2200 genomic DNA screen tape kit (5067-5366, 5067-5365; Agilent). Unless specified, all enzymes used for library preparation were purchased from NEB. A total of 300ng of gDNA was sheared using a Covaris S220 (Covaris), and processed for end-repair and A-tailing. Pre-annealed methylated adapters (synthesized by IDT, see table for sequences) were ligated in a thermocycler at 16°C overnight. Ligated fragments were cleaned using MinElute columns (28004; Qiagen), and the eluted ligated fragments were size selected on a 1% Agarose-EtBR gel, cleaned and bisulfite converted using the EZ DNA Methylation kit (5002; Zymo Research) and the following protocol: 55 cycles (95°C, 30sec; 50°C, 15 min), 4°C 10 min. The bisulfite converted fragments were cleaned using the manufacturer’s protocol and PCR amplified through 18 round with Roche HiFi Fast Start kit (3553361001; Sigma-Aldrich), and using PE1.0 and PE2.0 primers (synthesized by IDT, see table for sequences). The final libraries were cleaned with AmPure XP beads (A63881; Beckman Coulter), quantified with the Qubit High Sensitivity dsDNA kit and checked on the TapeStation 2200 (RRID:SCR_014994) with the High Sensitivity D1000 Assay (5067-5584,5067-5585; Agilent). The libraries were subjected to sequence on a HiSeq 2500 system (RRID:SCR_016383) (50-bp single-end) at the University of Michigan Advanced Genomics Core.

The methylation levels of intersected 100-bp CpG regions (i.e. regions covered by all samples) were used for Uniform Manifold Approximation and Projection for Dimension Reduction (UMAP) analysis, which was implemented by R *umap* package (v0.2.4.1) using the default parameters (https://CRAN.R-project.org/package=umap). The top 5000 variable regions were selected based on median absolute deviation (MAD) values and used to cluster the 36 samples by consensus clustering (k=5) [1].

***Identification of differentially methylated regions***

To identify the differentially methylated regions (DMRs), we used the DSS model implemented in the R *methylSig* package (v0.99.0) [2]. Common clinical variables, including sex, age, smoking status (smoker versus never) and stage (IV versus others), as well as HPV gene expression levels (CPM), were considered potential covariates in the general model. To identify the optimal set of covariates, we performed a backward selection process as previously described [3]. Briefly, a DSS model was first fit using methylation levels of 100-bp CpG regions (meth%) and the full set of covariates using the diff_dss_fit function: in the IMU versus HPV(-) and KRT versus HPV(-) comparisons, sex, age, smoking status and stage were included, while in IMU versus KRT and HPVint(+) vs HPVint(-) comparisons, HPV gene expression level was also included. In each iteration, the least relevant covariate was dropped if the number of its significantly associated regions (FDR < 0.05) was less than 0.1%, or if multiple covariates had zero significant regions. The models with the full set of covariates turned out to be optimal and were used for the DMR identification.

***RNA-seq analysis of previously published data***

Differential gene expression analysis (IMU versus KRT, IMU versus HPV(-), KRT versus HPV(-) and HPVint(+) versus HPVint(-)) for previously published RNA-seq data of the same 36 tumor samples (GSE74927) was performed using the edgeR v3.34.1 [4]. Bioconductor package with upper quartile normalization and the glmQLFit function. More information about the library preparation and preprocessing of the RNA-seq data is available at [5]. The covariates included in the design matrix were the same as above, including sex, age, smoking status (smoker versus never) and stage (IV versus others), as well as HPV gene expression levels (CPM) for HPV(+) sample comparisons, in order to be consistent with the DMR analysis, which also ensured a fair comparison for the following gene set enrichment results.

***Comparison with the methylation changes between tumor and normal samples in the TCGA HNSC cohort***

We visualized methylation levels at CpG sites that were both differentially methylated between IMU versus KRT in our cohort and the tumor versus normal in The Cancer Genome Atlas (TCGA) HNSC cohort. To do so, we downloaded methylation data from 528 HNSCC and 50 normal samples from TCGA (<https://www.cancer.gov/tcga>), which were measured by Illumina’s Infinium® HumanMethylation450 BeadChip. The differentially methylated probes (DMPs) between tumor and normal were identified using the eBayes method found in the R package *limma* [6]. This analysis was done with the M-values and the DMPs were selected based on a cutoff of FDR < 0.05 and absolute methylation difference ≥ 10%. Using Illumina’s accompanying annotation data (IlluminaHumanMethylation450kanno.ilmn12.hg19 [7]) to obtain the genomic locations of the BeadChip probes, the tumor versus normal DMPs that overlapped with DMRs between IMU and KRT were visualized using the *ComplexHeatmap* R package [8]. The proportion methylation (i.e. beta) values from all 18 HPV(+) samples were clustered using the default settings of ComplexHeatmap, namely using Euclidean distance measure and complete linkage as the clustering method. Both the columns and the rows of the heatmap were clustered using these methods. The heatmaps shown in Figure 5A and 5B were generated with this clustered order, and with the additional tumor and normal columns added on at the far right (i.e. not part of the clustering).

***Calculation of patient-level methylation at different genomic regions***

The overall methylation levels at particular genomic regions (upstream 2-kb of 5’UTR, 5’UTR exon, coding exon, first intron, internal intron, last intron, 3’UTR exon and the downstream 2-kb of 3’UTR, as well as the promoters, CpG island, repetitive regions and their up/down-stream 2kb regions) were calculated for individual samples using the *beta_profile_gene_centered.py* and *beta_profile_gene_centered.py* functions from the *CpGtools* python package [9]. The repetitive regions of the hg19 genome were retrieved from “repeatMasker” track (downloaded on Feb. 14, 2022) in UCSC Table Browser (https://genome.ucsc.edu/cgi-bin/hgTables). The average methylation level in each sample for each genomic region type, as well as across samples within individual subtypes, were generated, resulting in patient-wise methylation scores and genomic-region-specific group-wise methylation scores, respectively. Since the up- and down-stream (2-kb window) signals at CpG island, promoter and repetitive regions were comparable, we combined their up-/downstream regions when calculating the patient-wise methylation scores.

***Calculation of cancer cell-specific methylation levels***

To decipher the cancer cell-specific methylation level in each patient, we first performed cell decomposition analysis on RNA-seq data by EPIC [10] or on WGBS data by MethylCIBERSORT [11], using the default immune/fibroblast cell types included in each algorithm. The proportion of cancer cells in each patient was then estimated by extracting the predicted proportions of immune/fibroblast cells. With the estimated proportions of immune cells and tumor, as well as the methylation levels in the reference immune cells extracted from the atlas dataset (GSE186458) [12] as described above, we were able to estimate the cancer cell-specific methylation levels in each patient according to the following formula, assuming the overall methylation level (${meth}_{o}$) is the summation of the methylation level in each immune/fibroblast cell types (${proportion}_{i}\times{meth}_{i}$) and cancer cells (${proportion}_{t}\times{meth}_{t}$) in each sample:

$${{meth}_{t}}= \frac{{meth}_{o} - \sum_{i = 1}^{n} {proportion}_{i}\times{meth}_{i}}{{proportion}_{t}}$$

Since some immune cell types were not investigated in GSE186458, we estimated their methylation levels based on their closest cell types, i.e. using average methylation of ”Granulocytes” and “Monocytes” to estimate the methylation of “Macrophage” and “Dendritic cells”.

***Calculation of pathway scores***

Patient-wise expression pathway scores were calculated using the RNA-seq data for five selected representative gene sets as described in [5]. Specifically, we summarized the expression levels for the pre-defined gene sets which measure the adaptive immune response (“T-cell.score” from *T cell activation* [GO: 0042110]), HPV oncogene E6 activity (“E6.score” from E6 regulated genes derived from table 1 in Duffy et al [13], and cell differentiation (“keratinocyte.score” from [*keratinocyte differentiation* [GO: 0030216]](http://www.informatics.jax.org/vocab/gene_ontology/GO:0030216) , “Mesenchymal.score” from *mesenchymal differentiation* [GO:0048762], and “EMT.score” from cancer EMT markers defined in table 1 in Zeisberg *et al I* [14].

***MIRA score analysis***

MIRA generates a DNA methylation profile for each sample, which is then used to visualize the percent DNA methylation across the region set of interest as well as calculate a corresponding MIRA score. The MIRA score represents the aggregate regulatory activity of a region set, in which a higher MIRA score corresponds to a stronger dip in methylation and therefore greater activity. We calculated MIRA scores for the 36 HNSCC patients by examining the regulatory activity of eight region sets of interest: strong and weak enhancers in NHEK cells and six TFs taken from the most relevant cell line available (**Table S4**). The TFs were selected based on biological relevance to HNSCC and available good quality ChIP-seq data. ChIP-seq data were obtained from either ENCODE or Cistrome DB [15], and peaks taken from Cistrome were converted from hg38 to hg19 using CrossMap [16]. ChIP-seq data from the most relevant cell line to HNSCC that displayed good quality was chosen.

MIRA uses two user-defined parameters to calculate the score: region size and bin number. A region size of 10,000 bp and bin number of 11 was used for the strong and weak enhancer region sets, and a region size of 5,000 bp and bin number of 21 were used for all TF region sets. Lastly, the obtained MIRA scores were visualized with ggpubr (0.4.0) and analyzed by ANOVA with *p*-values < 0.00625 considered significant, to account for multiple testing of eight region sets.

**References**

1. Wilkerson, M.D.; Hayes, D.N. ConsensusClusterPlus: A Class Discovery Tool with Confidence Assessments and Item Tracking. *Bioinformatics* **2010**, *26*, 1572–1573.

2. Park, Y.; Figueroa, M.E.; Rozek, L.S.; Sartor, M.A. MethylSig: A Whole Genome DNA Methylation Analysis Pipeline. *Bioinformatics* **2014**, *30*, 2414–2422.

3. Qin, T.; Koneva, L.A.; Liu, Y.; Zhang, Y.; Arthur, A.E.; Zarins, K.R.; Carey, T.E.; Chepeha, D.; Wolf, G.T.; Rozek, L.S.; et al. Significant Association between Host Transcriptome-Derived HPV Oncogene E6* Influence Score and Carcinogenic Pathways, Tumor Size, and Survival in Head and Neck Cancer. *Head Neck* **2020**, *42*, doi:10.1002/hed.26244.

4. Robinson, M.D.; McCarthy, D.J.; Smyth, G.K. EdgeR: A Bioconductor Package for Differential Expression Analysis of Digital Gene Expression Data. *Bioinformatics* **2010**, *26*, 139–140.

5. Zhang, Y.; Koneva, L.A.; Virani, S.; Arthur, A.E.; Virani, A.; Hall, P.B.; Warden, C.D.; Carey, T.E.; Chepeha, D.B.; Prince, M.E.; et al. Subtypes of HPV-Positive Head and Neck Cancers Are Associated with HPV Characteristics, Copy Number Alterations, PIK3CA Mutation, and Pathway Signatures. *Clin. Cancer Res.* **2016**, *22*, 4735–4745.

6. Ritchie, M.E.; Phipson, B.; Wu, D.; Hu, Y.; Law, C.W.; Shi, W.; Smyth, G.K. Limma Powers Differential Expression Analyses for RNA-Sequencing and Microarray Studies. *Nucleic Acids Res.* **2015**, *43*, e47–e47.

7. Hansen IlluminaHumanMethylation450kanno. Ilmn12. Hg19: Annotation for Illumina’s 450k Methylation Arrays. *R package version 0.6. 0*.

8. Gu, Z.; Hübschmann, D. Make Interactive Complex Heatmaps in R. *Bioinformatics* **2021**, doi:10.1093/bioinformatics/btab806.

9. Wei, T.; Nie, J.; Larson, N.B.; Ye, Z.; Eckel-Passow, J.E.; Robertson, K.D.; Kocher, J.-P.A.; Wang, L. CpGtools: A Python Package for DNA Methylation Analysis. *Bioinformatics* **2021**, *37*, 1598–1599.

10. Racle, J.; Gfeller, D. EPIC: A Tool to Estimate the Proportions of Different Cell Types from Bulk Gene Expression Data. *Methods Mol. Biol.* **2020**, *2120*, doi:10.1007/978-1-0716-0327-7_17.

11. Chakravarthy, A.; Furness, A.; Joshi, K.; Ghorani, E.; Ford, K.; Ward, M.J.; King, E.V.; Lechner, M.; Marafioti, T.; Quezada, S.A.; et al. Pan-Cancer Deconvolution of Tumour Composition Using DNA Methylation. *Nat. Commun.* **2018**, *9*, 1–13.

12. Loyfer, N.; Magenheim, J.; Peretz, A.; Cann, G.; Bredno, J.; Klochendler, A.; Fox-Fisher, I.; Shabi-Porat, S.; Hecht, M.; Pelet, T.; et al. A Human DNA Methylation Atlas Reveals Principles of Cell Type-Specific Methylation and Identifies Thousands of Cell Type-Specific Regulatory Elements. *bioRxiv* 2022, 2022.01.24.477547.

13. Duffy, C.L.; Phillips, S.L.; Klingelhutz, A.J. Microarray Analysis Identifies Differentiation-Associated Genes Regulated by Human Papillomavirus Type 16 E6. *Virology* **2003**, *314*, doi:10.1016/s0042-6822(03)00390-8.

14. Zeisberg, M.; Neilson, E.G. Biomarkers for Epithelial-Mesenchymal Transitions. *J. Clin. Invest.* **2009**, *119*, doi:10.1172/JCI36183.

15. Zheng, R.; Wan, C.; Mei, S.; Qin, Q.; Wu, Q.; Sun, H.; Chen, C.-H.; Brown, M.; Zhang, X.; Meyer, C.A.; et al. Cistrome Data Browser: Expanded Datasets and New Tools for Gene Regulatory Analysis. *Nucleic Acids Res.* **2019**, *47*, D729–D735.

16. Zhao, H.; Sun, Z.; Wang, J.; Huang, H.; Kocher, J.P.; Wang, L. CrossMap: A Versatile Tool for Coordinate Conversion between Genome Assemblies. *Bioinformatics* **2014**, *30*, doi:10.1093/bioinformatics/btt730.
