# Supplementary material for: Whole-genome CpG-resolution DNA Methylation Profiling of HNSCC Reveals Distinct Mechanisms of Carcinogenesis for Fine-scale HPV+ Cancer Subtypes
Source: Cancer Res Commun. 2023 Aug 30;3(8):1701–15. doi: 10.1158/2767-9764.CRC-23-0009 (PMC10467604; doi:10.1158/2767-9764.CRC-23-0009)
Supplement: Supplementary Fig 1 — Companion figure showing significance results for main Figure 1D-F and Figure 2A. [file crc-23-0009-s07.docx]

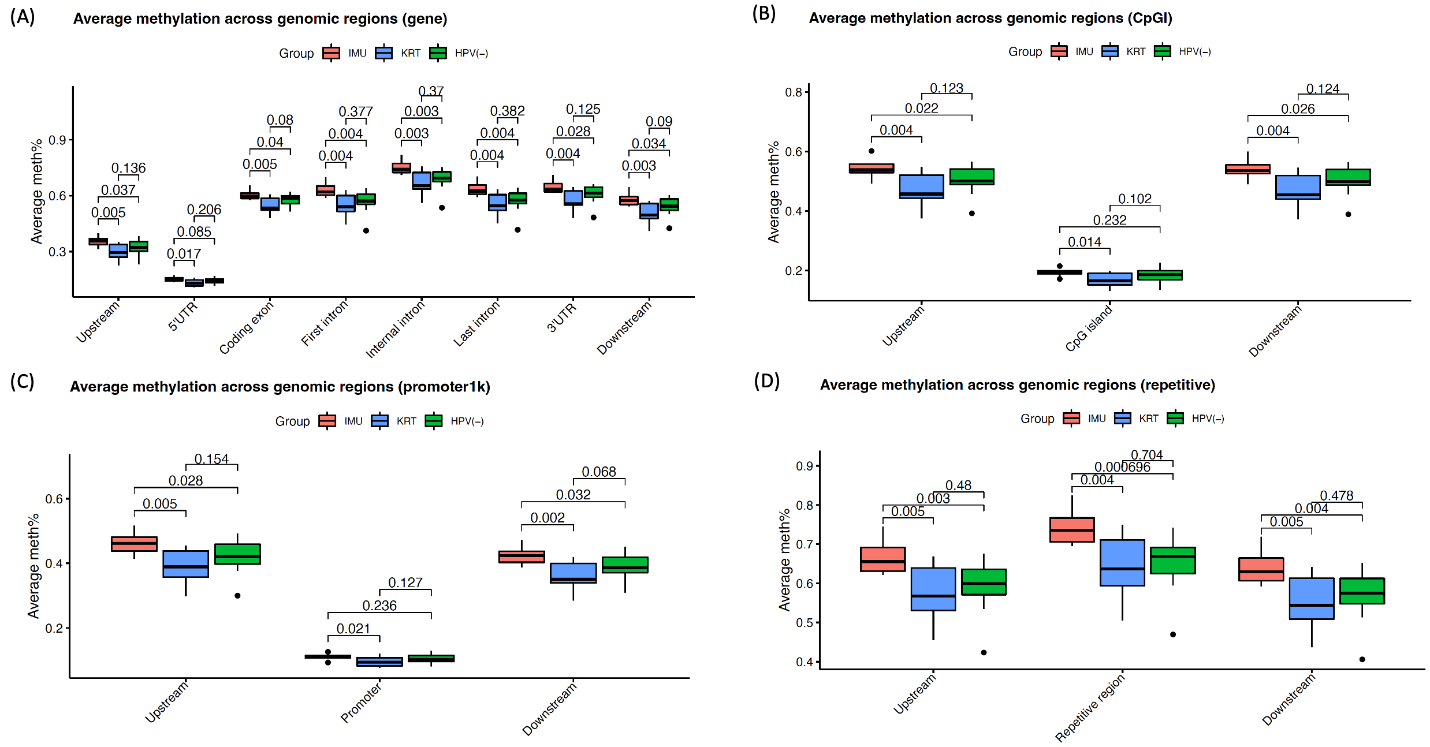


**Supplementary Figure S1. Companion figure showing significance results for main Figure 1D-F and Figure 2A.** Comparisons of average methylation at genic (A), promoter (B), CpG island (C) and repetitive region (D). Samples were grouped by HPV(+) subtypes (IMU and KRT) and HPV(-) tumors. Wilcoxon Rank-Sum p values are displayed.
