# Supplementary material for: Whole-genome CpG-resolution DNA Methylation Profiling of HNSCC Reveals Distinct Mechanisms of Carcinogenesis for Fine-scale HPV+ Cancer Subtypes
Source: Cancer Res Commun. 2023 Aug 30;3(8):1701–15. doi: 10.1158/2767-9764.CRC-23-0009 (PMC10467604; doi:10.1158/2767-9764.CRC-23-0009)
Supplement: Supplementary Fig 2 — Average methylation of tumors and normal epithelial cells at gene body (A), promoter (B), CpG island (C) and repetitive region (D). Samples were grouped by HPVor HPV integration status. [file crc-23-0009-s08.docx]

**Supplementary Figure S2.** Average methylation of tumors and normal epithelial cells at gene body (A), promoter (B), CpG island (C) and repetitive region (D). Samples were grouped by HPVor HPV integration status.
