# Supplementary material for: Whole-genome CpG-resolution DNA Methylation Profiling of HNSCC Reveals Distinct Mechanisms of Carcinogenesis for Fine-scale HPV+ Cancer Subtypes
Source: Cancer Res Commun. 2023 Aug 30;3(8):1701–15. doi: 10.1158/2767-9764.CRC-23-0009 (PMC10467604; doi:10.1158/2767-9764.CRC-23-0009)
Supplement: Supplementary Fig 3 — Comparison of locations of positive versus negative cis-eQTMs that overlap with DMRs for: IMU vs HPV(-) (1st row), IMU vs KRT (2nd row), and KRT vs HPV(-) (3rd row) of each panel. (A) CpG island annotations; (B) genic annotations. 1st and 2nd columns are hypermethylated in the first comparison group; 3rd and 4th columns are hypomethylated in the first comparison group. Negative eQTMs are in the 1st and 3rd columns, while positive eQTMs are in the 2nd and 4th columns. [file crc-23-0009-s09.docx]

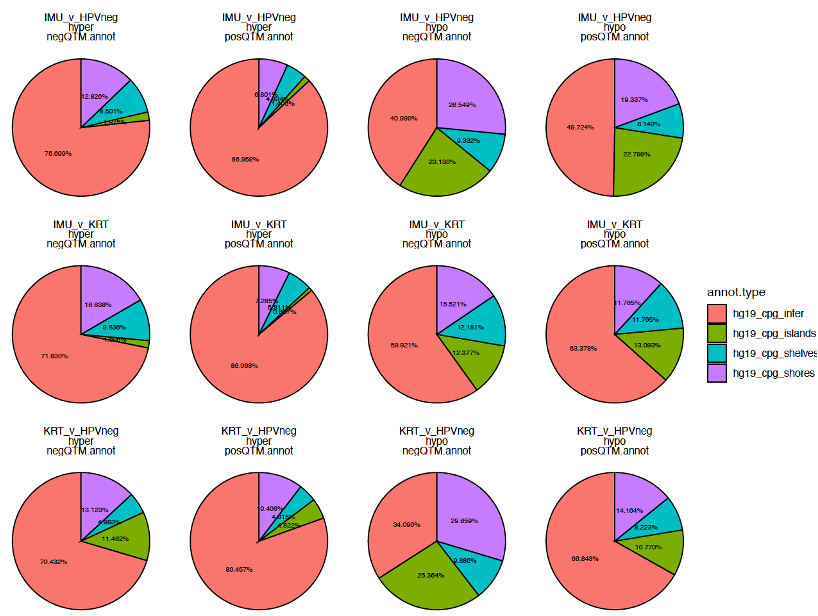

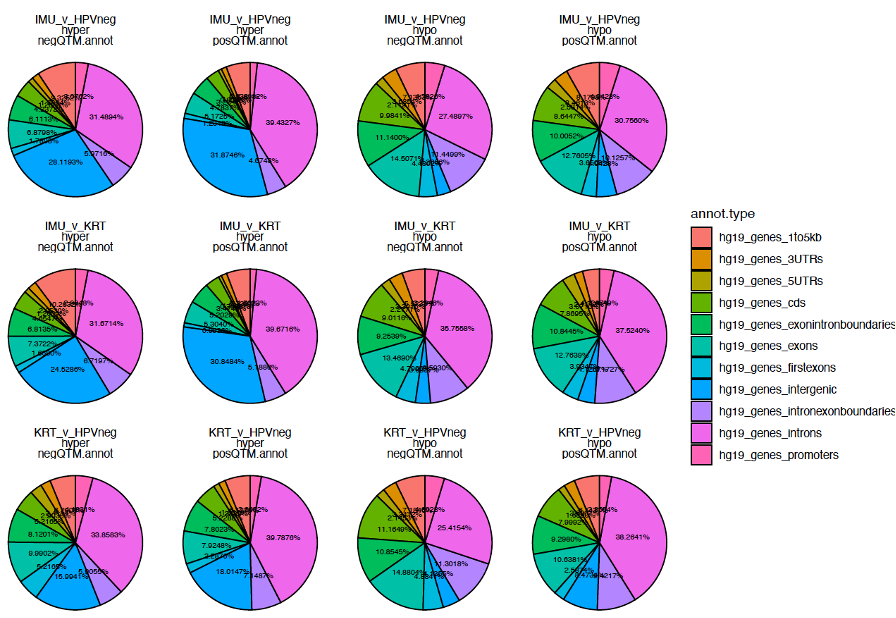


**Supplementary Figure S3.** Comparison of locations of positive versus negative cis-eQTMs that overlap with DMRs for: IMU vs HPV(-) (1^st^ row), IMU vs KRT (2^nd^ row), and KRT vs HPV(-) (3^rd^ row) of each panel. (A) CpG island annotations; (B) genic annotations. 1^st^ and 2^nd^ columns are hypermethylated in the first comparison group; 3^rd^ and 4^th^ columns are hypomethylated in the first comparison group. Negative eQTMs are in the 1^st^ and 3^rd^ columns, while positive eQTMs are in the 2^nd^ and 4^th^ columns.
