# Supplementary material for: Whole-genome CpG-resolution DNA Methylation Profiling of HNSCC Reveals Distinct Mechanisms of Carcinogenesis for Fine-scale HPV+ Cancer Subtypes
Source: Cancer Res Commun. 2023 Aug 30;3(8):1701–15. doi: 10.1158/2767-9764.CRC-23-0009 (PMC10467604; doi:10.1158/2767-9764.CRC-23-0009)
Supplement: Supplementary Fig 4 — Patient-level methylation at repetitive regions, stratified by disease stage (A), by subtype and HPV status across genome (B) or at different genomic regions (C). [file crc-23-0009-s10.docx]

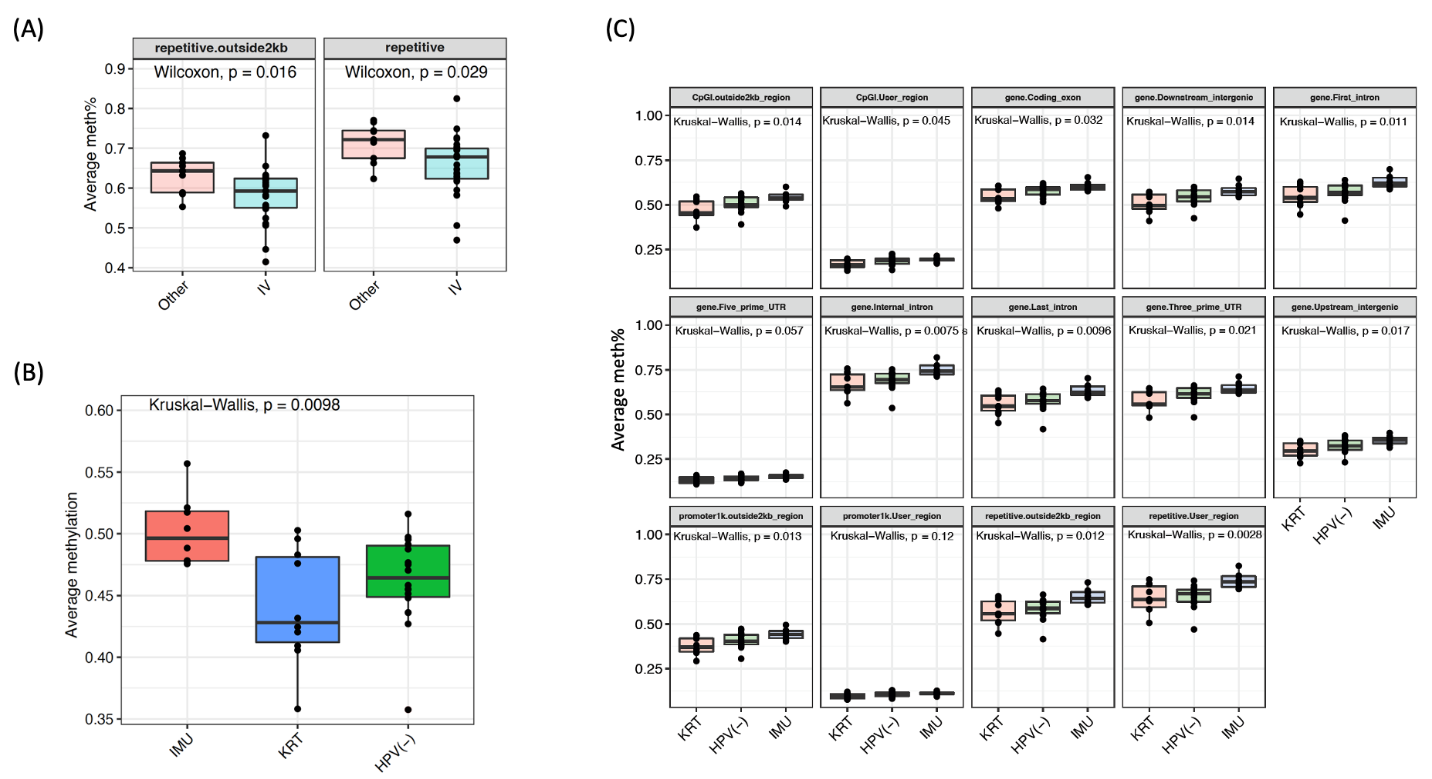


**Supplementary Figure S4.** Patient-level methylation at repetitive regions, stratified by disease stage (A), by subtype and HPV status across genome (B) or at different genomic regions (C).
