# Supplementary material for: Whole-genome CpG-resolution DNA Methylation Profiling of HNSCC Reveals Distinct Mechanisms of Carcinogenesis for Fine-scale HPV+ Cancer Subtypes
Source: Cancer Res Commun. 2023 Aug 30;3(8):1701–15. doi: 10.1158/2767-9764.CRC-23-0009 (PMC10467604; doi:10.1158/2767-9764.CRC-23-0009)
Supplement: Supplementary Fig 5 — Correlate GSE testing results from mRNA expression and DNA methylation in the exon, intron regions and keratinization genes visualization. [file crc-23-0009-s11.docx]

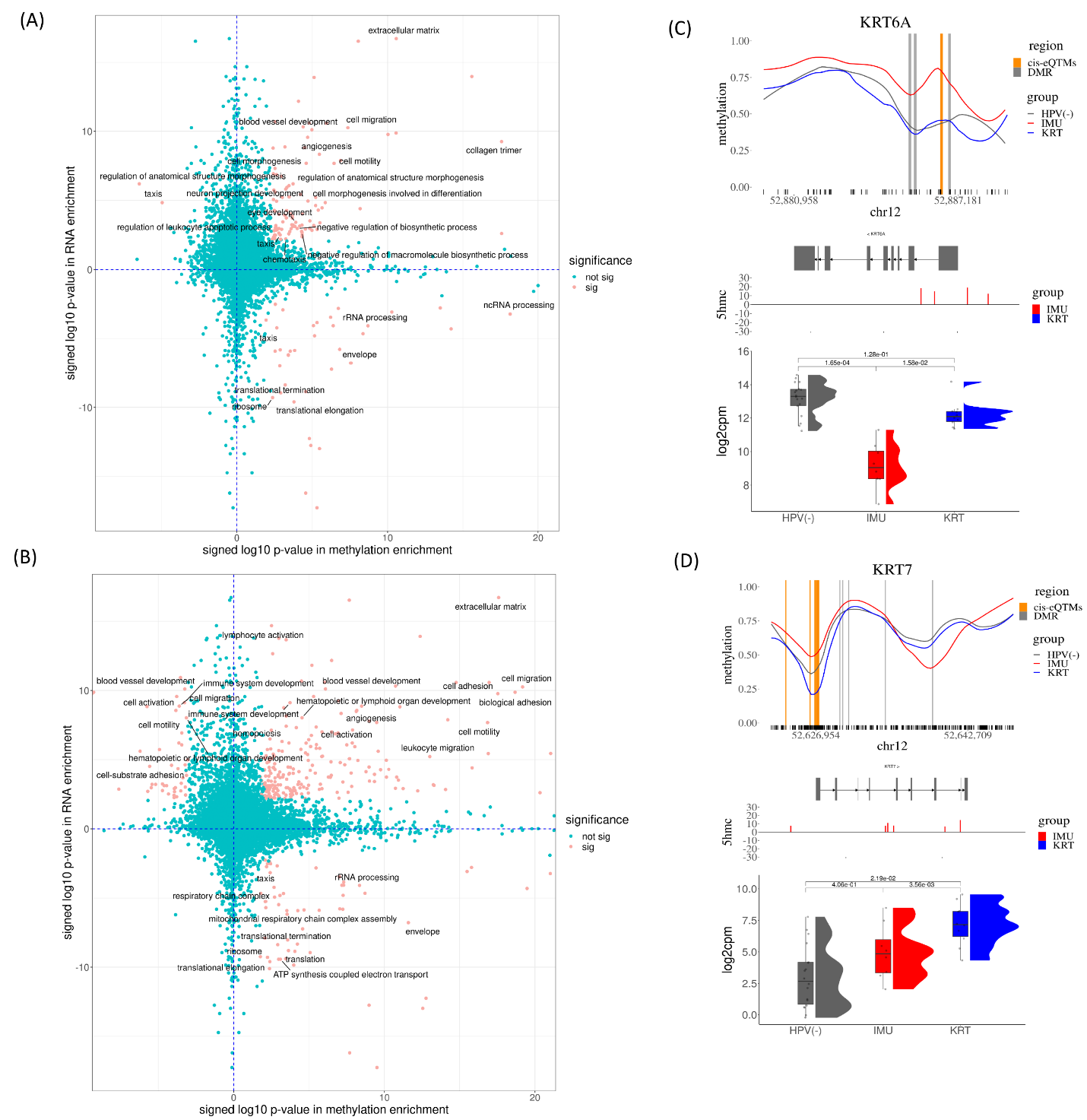


**Supplementary Figure S5.** Correlate GSE testing results from mRNA expression and DNA methylation in the exon, intron regions and keratinization genes visualization. (A) scatter plot for directional RNA and exon regions’ DNA methylation enrichment testing. Right quadrants of methylation testing represent all significant hypermethylation regions; the top quadrants of the RNA enrichment represent IMU enriched GO terms. Color: terms both significant with FDR<0.05. (B) scatter plot for directional RNA and intron regions’ DNA methylation enrichment testing. (C-D) specific genes’ visualization from keratinization GO terms.
