# Supplementary material for: Whole-genome CpG-resolution DNA Methylation Profiling of HNSCC Reveals Distinct Mechanisms of Carcinogenesis for Fine-scale HPV+ Cancer Subtypes
Source: Cancer Res Commun. 2023 Aug 30;3(8):1701–15. doi: 10.1158/2767-9764.CRC-23-0009 (PMC10467604; doi:10.1158/2767-9764.CRC-23-0009)
Supplement: Supplementary Fig 6 — Methylation and expression visualization of the genes selected from the 108 genes previously reported as differentially methylated in HPV(+) compared to HPV(-) HNSCC. [file crc-23-0009-s12.docx]

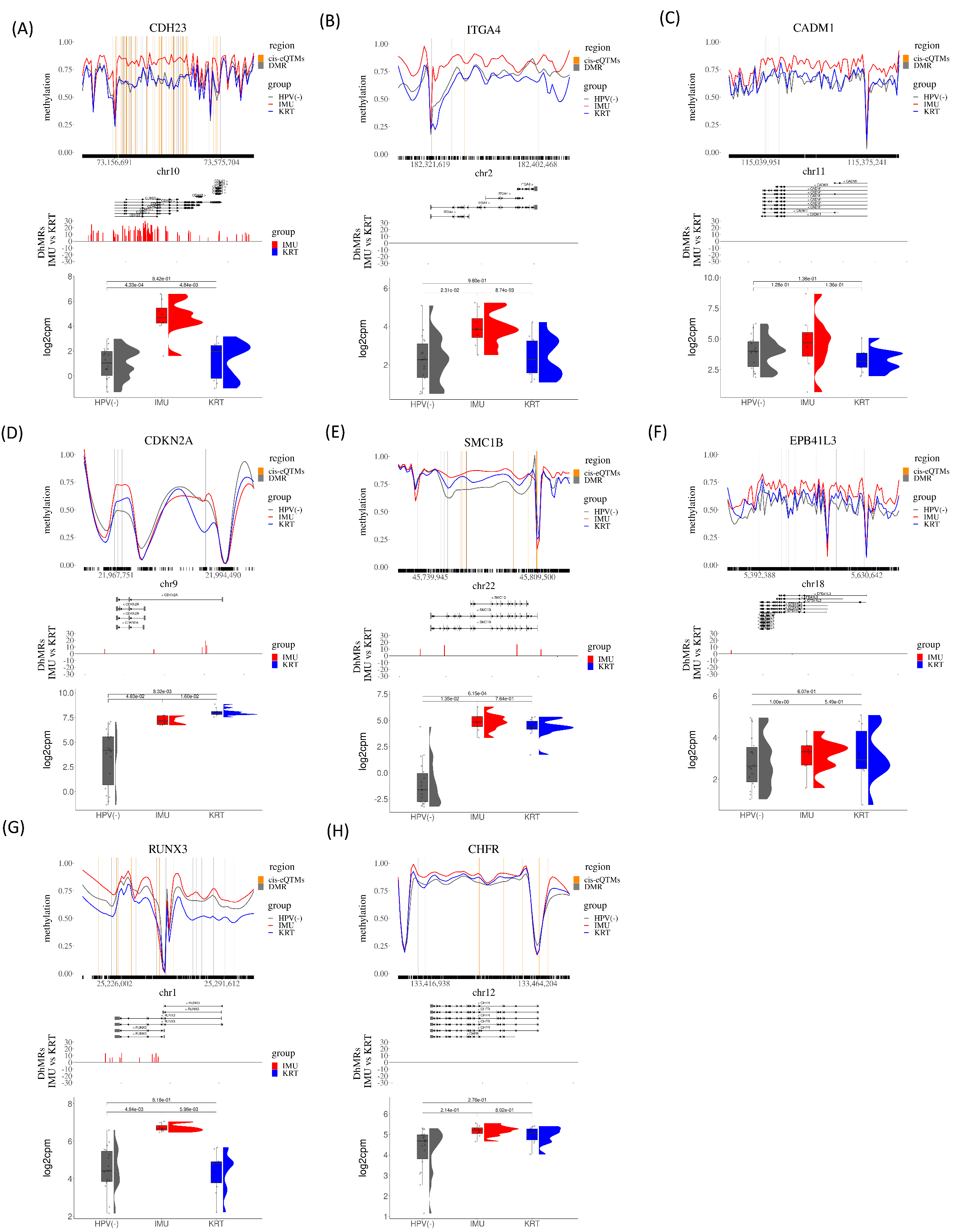


**Supplementary Figure S6.** Methylation and expression visualization of the genes selected from the 108 genes previously reported as differentially methylated in HPV(+) compared to HPV(-) HNSCC. (A-C) Genes related to cell adhesion. (D-F) genes related to cell cycle and apoptosis (G) RUNX family gene (H) representative tumor suppressor gene- CHFR. The regions highlighted are representing DMRs from either IMU or KRT versus HPV(-) and overlapped eQTM regions.
