# Supplementary material for: Whole-genome CpG-resolution DNA Methylation Profiling of HNSCC Reveals Distinct Mechanisms of Carcinogenesis for Fine-scale HPV+ Cancer Subtypes
Source: Cancer Res Commun. 2023 Aug 30;3(8):1701–15. doi: 10.1158/2767-9764.CRC-23-0009 (PMC10467604; doi:10.1158/2767-9764.CRC-23-0009)
Supplement: Supplementary Fig 7 — Methylation and expression profiles of the eleven genes that are hypermethylated in TCGA HNSC tumor vs normal, and hypermethylated in KRT vs IMU shown in Figure 5C. [file crc-23-0009-s13.docx]

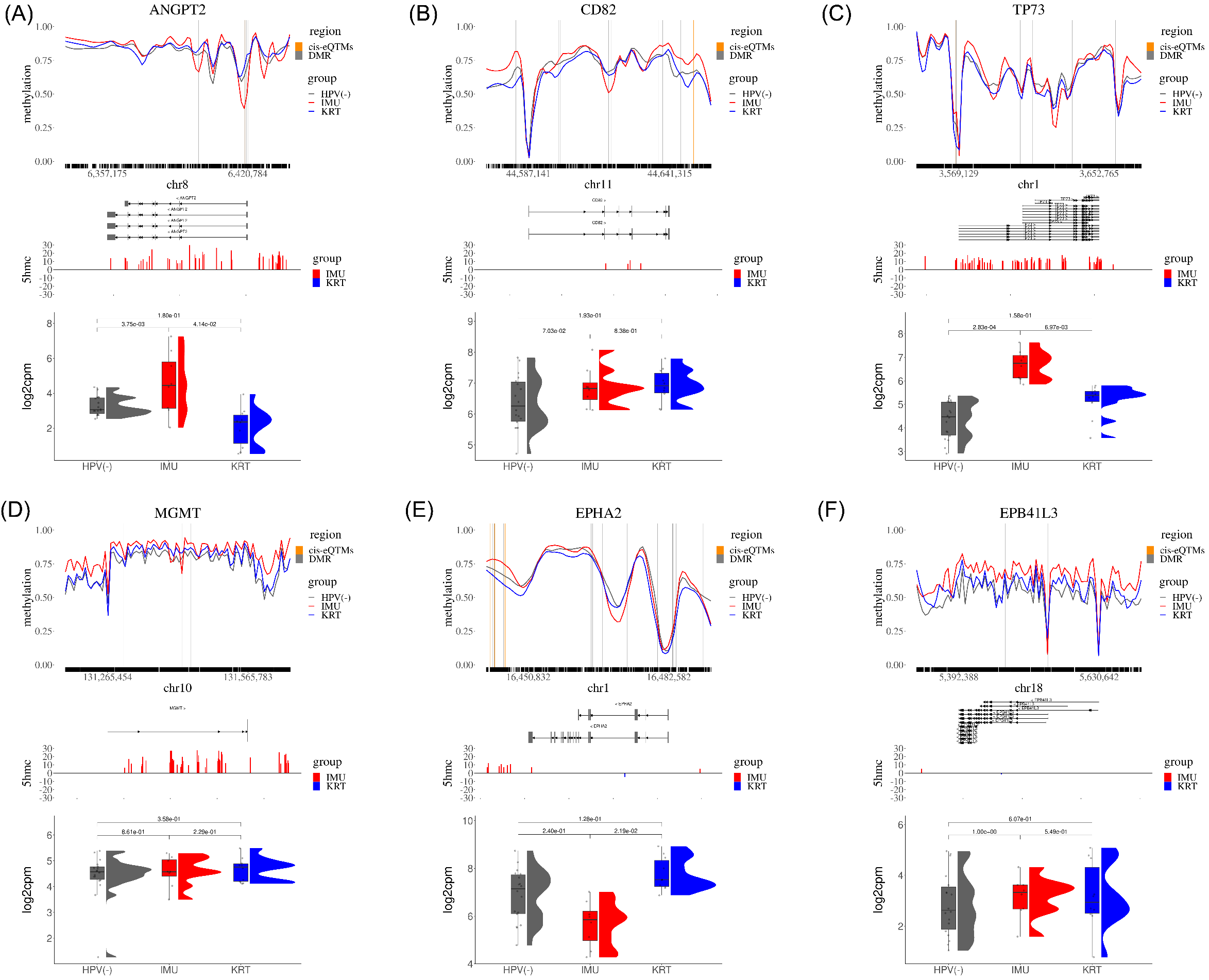

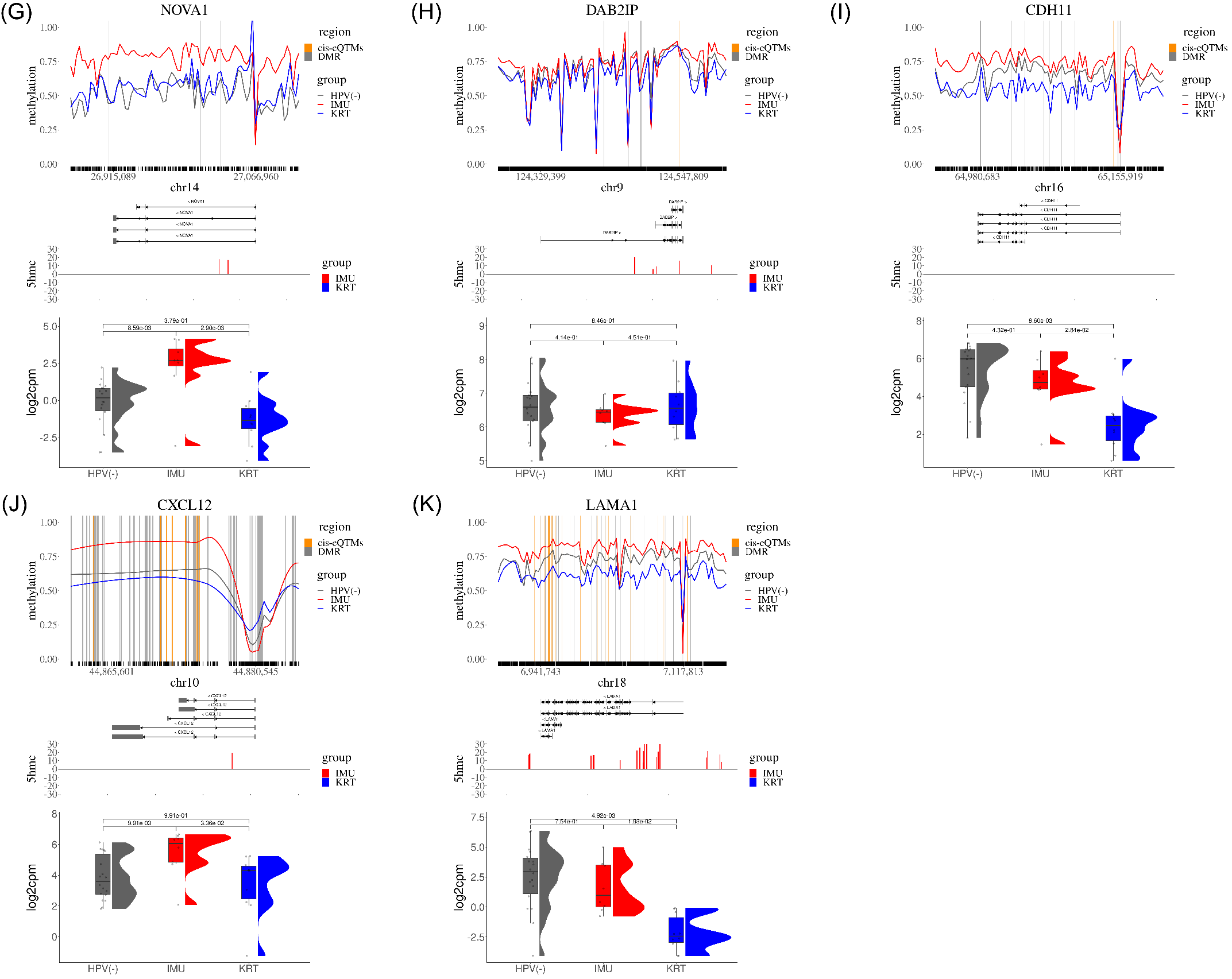


**Supplementary Figure S7.** Methylation and expression profiles of the eleven genes that are hypermethylated in TCGA HNSC tumor vs normal, and hypermethylated in KRT vs IMU shown in Figure 5C.
