# Supplementary material for: Whole-genome CpG-resolution DNA Methylation Profiling of HNSCC Reveals Distinct Mechanisms of Carcinogenesis for Fine-scale HPV+ Cancer Subtypes
Source: Cancer Res Commun. 2023 Aug 30;3(8):1701–15. doi: 10.1158/2767-9764.CRC-23-0009 (PMC10467604; doi:10.1158/2767-9764.CRC-23-0009)
Supplement: Supplementary Fig 8 — (A-D) MIRA profiles of strong and weak enhancers in the NHEK cell line (A), JunD binding sites in the K562 cell line (B), NFKB binding sites in the GM10847 cell line, and STAT3 binding sites in the HeLaS3 cell line (D) Samples are colored by HPV tumor subtype. (E) Correlations between CTCF MIRA scores and Strong Enhancer MIRA scores. (F) Box plot of MIRA scores separated by subtype for EZH2 binding sites and JunB binding sites from NHEK and K562 cell lines, respectively. (G) Correlations between JunD MIRA scores and gene expression-based keratinization scores. All scatter plots separate subtype by color and display the Pearson correlation coefficient (R) along with the corresponding p-value. [file crc-23-0009-s14.docx]

**Supplementary Figure S8. (A-D) MIRA profiles of strong and weak enhancers in the NHEK cell line (A), JunD binding sites in the K562 cell line (B), NFKB binding sites in the GM10847 cell line, and STAT3 binding sites in the HeLaS3 cell line (D) Samples are colored by HPV tumor subtype.** (E) Correlations between CTCF MIRA scores and Strong Enhancer MIRA scores. (F) Box plot of MIRA scores separated by subtype for EZH2 binding sites and JunB binding sites from NHEK and K562 cell lines, respectively. (G) Correlations between JunD MIRA scores and gene expression-based keratinization scores. All scatter plots separate subtype by color and display the Pearson correlation coefficient (R) along with the corresponding p-value.
